# Predictive Factors of Renal Recovery and Progression to End-Stage Kidney Disease in Patients With Antineutrophil Cytoplasmic Autoantibody-Associated Vasculitis With Severe Kidney Disease

**DOI:** 10.1016/j.ekir.2024.02.1431

**Published:** 2024-03-06

**Authors:** Marta Casal Moura, Dalia Zubidat, Marc Patricio Liebana, Sanjeev Sethi, Maria Jose Soler, Ladan Zand, Fernanda G. dos Santos, Luca Nardelli, Juan Leon-Roman, Ciria Sousa, Kenneth J. Warrington, Ulrich Specks, Fernando C. Fervenza

**Affiliations:** 1 Division of Pulmonary and Critical Care Medicine, Department of Medicine, Mayo Clinic College of Medicine and Science, Rochester, Minnesota, USA; 2 Division of Nephrology and Hypertension, Department of Medicine, Mayo Clinic College of Medicine and Science, Rochester, Minnesota, USA; 3 Servicio de Nefrologia, Centro de Referencia en Enfermedad Glomerular Compleja del Sistema Nacional de Salud (CSUR), Hospital Universitari Vall d'Hebron, Barcelona, Catalunya, Spain; 4 Department of Laboratory Medicine and Pathology, Mayo Clinic, Rochester, Minnesota, USA; 5 Division of Rheumatology, Department of Medicine, Mayo Clinic College of Medicine and Science, Rochester, Minnesota, USA

**Keywords:** ANCA, chronicity changes, glomerulonephritis, kidney biopsy, plasma exchange, vasculitis

## Abstract

**Introduction:**

A significant number of patients with antineutrophil cytoplasmic antibodies (ANCA)- associated vasculitis (AAV) with glomerulonephritis (AAV-GN) still progress to end-stage kidney disease (ESKD, estimated glomerular filtration rate [eGFR] <15 ml/min per 1.73 m^2^) despite advances in remission-induction treatment.

**Methods:**

This is a retrospective cohort study on myeloperoxidase (MPO)-ANCA or proteinase 3 (PR3)-ANCA positive patients with AAV (microscopic polyangiitis, MPA; or granulomatosis with polyangiitis, GPA) and eGFR <15 ml/min per 1.73 m^2^ or ESKD at presentation. Renal recovery, dialysis discontinuation, and persistence of ESKD after standard remission-induction, with or without the use of plasma exchange (PLEX) were analyzed.

**Results:**

We analyzed 166 patients with biopsy-proven active AAV-GN and eGFR <15 ml/min per 1.73 m^2^ at the time of diagnosis. Patients received glucocorticoids with cyclophosphamide (CYC) (*n* = 84) or with rituximab (RTX) (*n* = 72) for remission-induction, and 49 received PLEX. The predictors of renal recovery were erythrocyte sedimentation rate, serum creatinine (SCr) at diagnosis, and minimal or mild chronicity changes. We further analyzed 71 patients who started dialysis with or without PLEX within 4 weeks of AAV-GN diagnosis. The predictors of dialysis discontinuation were minimal chronicity changes in kidney biopsy at diagnosis (odds ratio = 6.138; 95% confidence interval [CI]: 1.389–27.118; *P* = 0.017). Predictors of persistence of ESKD within 12 months included higher SCr at diagnosis (incidence rate ratio [IRR] = 1.086; 95% CI: 1.005–1.173; *P* = 0.037), and moderate (IRR = 3.797; 95% CI: 1.090–13.225; *P* = 0.036), or severe chronicity changes in kidney biopsy (IRR = 5.883; 95% CI: 1.542–22.439; *P* =0.009).

**Conclusion:**

In our cohort, kidney recovery, dialysis discontinuation, and persistence of ESKD in patients with AAV-GN and eGFR <15 ml/min per 1.73 m^2^ depended on SCr and histologic findings on kidney biopsies at the time of diagnosis and was not affected by the addition of PLEX.

## Graphical abstract

**Figure:**
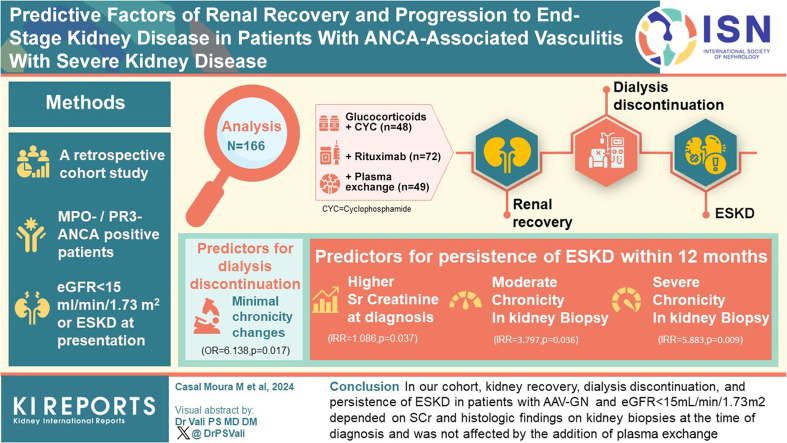

The risk of progression to ESKD in patients with AAV and AAV-GN remains high^1^, ^2^, ^3^, ^4^, ^5^, ^6^ with up to 25% of all patients evolving to ESKD at 5 years.^7^, ^8^, ^9^ Improvements in the rates of ESKD over the past decades have been mainly attributed to early diagnosis rather than to therapeutic interventions.^4^^,^^5^^,^^10^ SCr levels or kidney biopsy findings at diagnosis have been proposed as the main predictors of progression to ESKD.^9^^,^^11^^,^^12^

However, the outcomes of patients with the most advanced impairment of renal function, that is, those with ESKD (eGFR <15 ml/min per 1.73 m^2^) are difficult to estimate from the available literature. Patients with more advanced degrees of kidney dysfunction were enrolled in the RITUXVAS (RTX vs. CYC in AAV) trial.^13^^,^^14^ At enrollment, patients in the RTX arm (*n* = 33) had a median eGFR of 20 ml/min per 1.73 m^2^, whereas patients in the CYC group (*n* = 11) had a median eGFR of 12 ml/min per 1.73 m^2^. This trial showed that 76% in the RTX arm and 82% in the CYC arm had sustained remission (*P* = 0.68).^13^^,^^14^ However, outcomes for patients with eGFR <15 ml/min per 1.73 m^2^ are not shown. In the RAVE (RTX in AAV) trial, patients with a SCr >4 mg/dl at baseline were excluded. Nevertheless, eGFR was <30.0 ml/min per 1.73 m^2^ in (*n* = 18) 35% randomized to the RTX group, and in (*n* = 14) 27% of those randomized to the CYC group. A *post hoc* analysis showed that those patients responded similarly to RTX and CYC (75% in the RTX group and 77% in the CYC/azathioprine group achieved complete remission at some point after remission-induction treatment).^15^, ^16^, ^17^ Similar to the RITUXVAS trial, outcomes of patients with eGFR <15 ml/min per 1.73 m^2^ were not reported. Neither of these trials showed a difference in treatment response between the respective treatments in patients with kidney involvement.^13^, ^14^, ^15^, ^16^^,^^18^ An observational study of 37 patients with eGFR <20.0 ml/min per 1.73 m^2^ found no difference in remission, renal recovery from ESKD, or death between treatment with RTX and glucocorticoids, with or without CYC (25 vs. 12, respectively).^17^ The outcomes of patients with eGFR <15 ml/min per 1.73 m^2^ were not described.

Until recently, the use of PLEX was considered standard-of-care for patients with AAV-GN and severe kidney involvement, based on results of the MEPEX (methylprednisolone versus plasma exchange) trial in 2007^19^ that showed PLEX to be of benefit to avoid the progression to ESKD by 12 months (hazard ratio: 0.47; 95% CI: 0.24–0.91; *P* = 0.03).^19^ This effect was lost after that time point.^20^ However, the PEXIVAS (plasma exchange and glucocorticoid dosing in the treatment of AAV) trial, the largest randomized controlled trial ever conducted in patients with AAV, showed no benefit of adding PLEX to remission-induction regimens, even in patients with SCr >5.6 mg/dl (hazard ratio: 0.77; 95% CI: 0.53–1.11).^21^ Nevertheless, a recent meta-analysis suggested that PLEX might benefit patients with SCr >5.7 mg/dl.^22^^,^^23^ The results of this meta-analysis were derived from studies performed over 40 years and did not take into account the role of kidney biopsy findings, nor the impact of novel treatments in avoiding progression to ESKD.^24^

To provide information about patients with the most severe form of kidney impairment caused by active AAV-GN (i.e., eGFR <15 ml/min per 1.73m^2^) we conducted the present study.

## Methods

### Study Design

**Figure 1:**
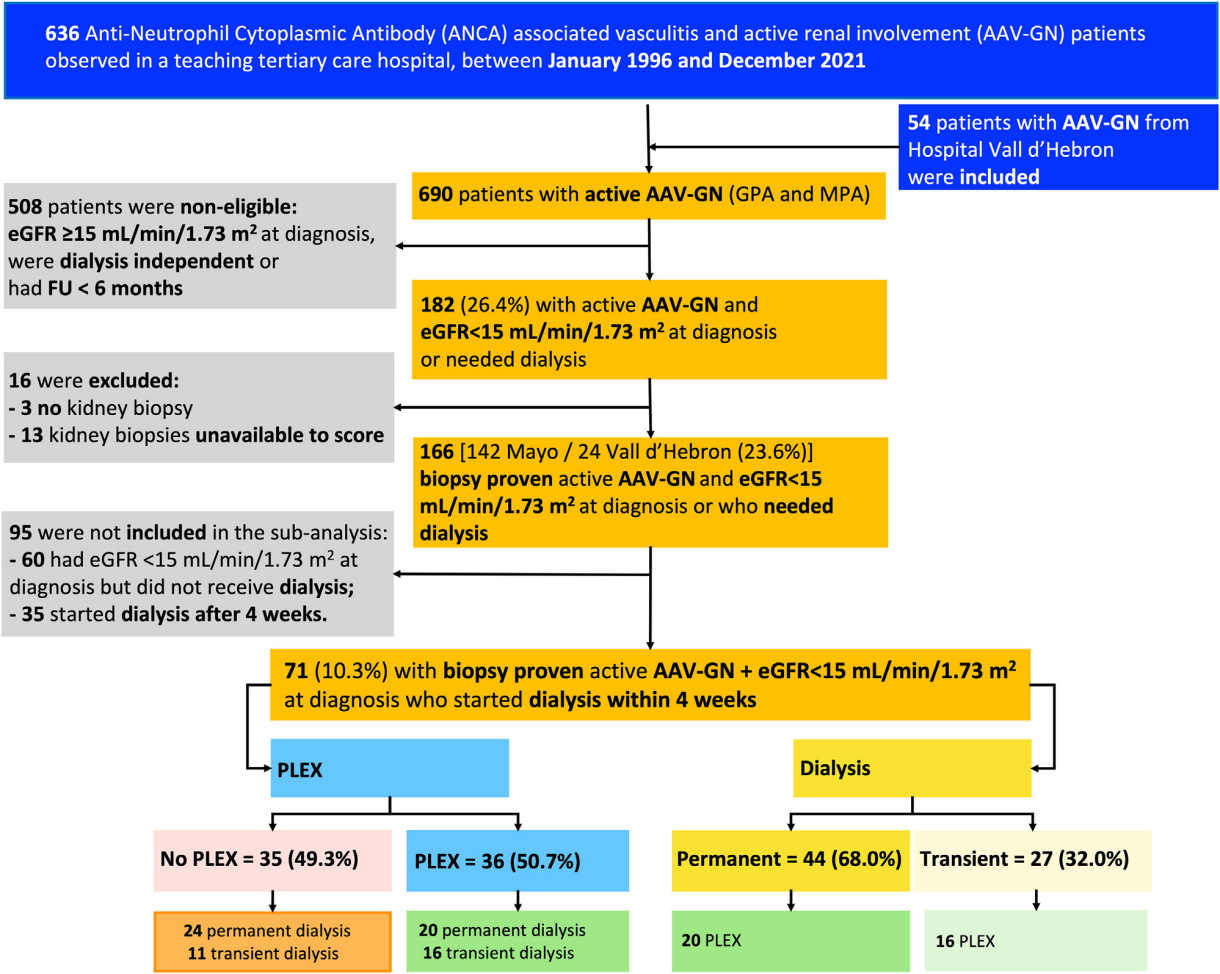
STROBE flowchart for the selection of the patient with active antineutrophil cytoplasmic antibodies (ANCA)-associated vasculitis (AAV) with glomerulonephritis (AVV-GN) and eGFR <15 ml/min per 1.73 m^2^ at diagnosis. Active renal involvement was defined by the presence of either (i) active, biopsy-proven, pauci-immune glomerulonephritis; (ii) red blood cell casts on urine microscopy; or (iii) rise in serum creatinine (SCr) >30% (or >25% decline in creatinine clearance) attributed to active vasculitis. Patients were grouped according to the addition of PLEX to remission-induction and according to the need of dialysis. eGFR, estimated glomerular filtration rate; FU, follow-up; GPA, granulomatosis with polyangiitis; MPA, microscopic polyangiitis; PLEX, plasma exchange.

All consecutive patients with AAV-GN evaluated at Mayo Clinic from January 1, 1996 to December 31, 2021 and all consecutive patients with AAV-GN evaluated at Vall d’Hebron from January 1, 2009 to December 31, 2021 were integrated in this study (Figure 1).

### Patient Characteristics

All data were abstracted retrospectively from electronic medical records and included demographic characteristics, comorbidities, laboratory findings, biopsy results, therapies, and outcomes. The date of the renal involvement diagnosis was registered for the calculation of follow-up times, time to dialysis and outcome time points. The Birmingham Vasculitis Activity Score for Wegener's Granulomatosis (BVAS/WG) was used to quantify disease activity at presentation and during the follow-up.^25^

MPO-ANCA or PR3-ANCA positive patients with newly diagnosed AAV or relapsing disease with active renal involvement and fulfilling the American College of Rheumatology criteria for GPA and the Chapel Hill consensus definitions for GPA and MPA were included (Figure 1).^26^, ^27^, ^28^ Kidney involvement was defined by the presence of the following: (i) active, biopsy-proven, pauci-immune glomerulonephritis; and (ii) eGFR <15 ml/min per 1.73 m^2^ at diagnosis of AAV-GN or dialysis initiation within 4 weeks of presentation were included, used as an objective criteria for eminent kidney failure. Patients with positive antiglomerular basement membrane, eosinophilic GPA, AAV without evidence of glomerulonephritis, or who were MPO-ANCA or PR3-ANCA negative were excluded.

### Renal Function Assessment

eGFR was calculated using the Chronic Kidney Disease Epidemiology Collaboration equation^29^^,^^30^ and was recorded at baseline, 6, 12, 18, and 24 months of follow-up, if available.

### Remission-Induction Therapies and Addition of PLEX to Standard Remission-Induction

Indication for remission-induction and treatment used were not defined by a preestablished protocol. Patients who received CYC (oral, 2 mg/kg/d for 6 months, adjusted for eGFR and age) followed a strict clinical practice protocol of laboratory monitoring, every 1 to 2 weeks for early detection of leukopenia driving dose adjustments. CYC i.v. regimens were rarely used. Patients who received RTX (i.v. 375 mg/m^2^ of body surface area once weekly for 4 weeks, or 1 g 2 weeks apart) had absolute B-cell counts measured at intervals determined by the treating physician. Only 6 patients treated at the Vall d'Hebron hospital received RTX in combination with CYC. Tapering of glucocorticoids was not performed following a preestablished protocol.

The decision to use PLEX was determined by the treating physician. The replacement fluid used for PLEX was 5% albumin with 3 units of fresh frozen plasma as the final portion in the presence or high risk of bleeding. The apheresis devices used during the study period to perform the plasma exchanges were the TerumoBCT COBE Spectra, TerumoBCT Spectra Optia, and the Fresenius KABI Fenwal Amicus. At Mayo Clinic, the anticoagulant consisted of acid citrate dextrose solution A with the addition of 10,000 units of heparin in patients without bleeding risk, and acid citrate dextrose solution A only in those with bleeding risk. At hospital Vall d’Hebron, the anticoagulant consisted of acid citrate dextrose solution A or low molecular weight heparin in patients without bleeding risk. A 1 plasma volume exchange were planned daily for 7 to 14 days with the apheresis devices calculating the volume to be exchanged based upon a hematocrit obtained within 24 hours of the procedure.^31^ As per our practice RTX was not given within the 48 hours prior to receiving a PLEX treatment.

### Outcomes Assessment

Remission was defined by a BVAS/WG of 0, independent of the dose of prednisone. Relapse was defined by an increase of BVAS/WG ≥1, that resulted in therapy changes (increases in doses of maintenance-remission therapy or the start of a new remission-induction cycle). The number of relapses after achievement of remission, type of relapse (major or minor), and the organ involvement (renal vs. nonrenal) were recorded. Renal recovery was defined as improvement of eGFR >15 ml/min per 1.73 m^2^. ESKD was defined as persistent eGFR ˂15 ml/min per 1.73 m^2^ or the need of renal replacement therapy (RRT). These outcomes were evaluated in the interval of 12 months. Dialysis discontinuation was evaluated in detail in patients who needed RRT, and early discontinuation was assumed if it happened within the first 4 weeks.

The primary aim of our study was to evaluate the incidence of eGFR <15 ml/min per 1.73 m^2^ or ESKD defined as the need of permanent RRT or kidney transplant, within 12 months. Secondary aims were the following: (i) identify predictive factors for renal recovery and ESKD in patients presenting with eGFR <15 ml/min per 1.73 m^2^; (ii) evaluate the impact of adding PLEX to standard remission-induction therapy for the acute renal outcomes in patients with eGFR <15 ml/min per 1.73 m^2^, with or without the need of RRT within 4 weeks of diagnosis. We highlighted the analysis on this time period to reflect patient outcomes during the most acute phase of the disease. We believe these patients are the ones that may benefit most from PLEX, because this is an intervention with presumably fast, but short-lived biologic effects.

### Statistical Analysis

Categorical variables were presented as count (percents) whereas continuous variables were presented as mean (SD) if they were normally distributed as determined by Shapiro-Wilk test, or as median (interquartile range [IQR]) and the IQR is reported as a range (reporting Q1 and Q3) if nonnormal. For comparisons of categorical variables between groups, the Pearson’s chi-square test was used if the number of elements in each cell was ≥5; Fisher exact test was used otherwise. For comparison of continuous variables between groups, an unpaired *t*-test for independent samples was used for distributions consistent with normality as above, and the Mann-Whitney U test was used otherwise.

Logistic regression models were developed to examine the predictive role of the baseline clinical characteristics for ESKD, renal recovery, and transient dialysis. The Kaplan-Meier method was used to assess cumulative incidence of ESKD within 12 months, the log-rank test to assess the differences in the time-to-event, and Cox proportional hazards regression models were used to determine predictive factors for permanent dialysis within 12 months. The IRR with a 95% CI was reported when appropriate.^32^ The time-to-event was recorded in months expressed to the centesimal place and it was not discretized. The outcome occurrence was analyzed in a determined time point (Supplementary Methods). IBM SPSS Statistics for MacOS, version 27 (IBM, Armonk, NY) was used for all data analysis.

## Results

### Patient Characteristics and Clinical Outcomes

#### Clinical Characteristics

Of the 690 patients with AAV evaluated during the study period (636 from the Mayo Clinic and 54 from Vall d’Hebron), only 38 patients were excluded due to no follow-up visit; active renal disease with eGFR <15 ml/min per 1.73 m^2^ was documented in 182 (26.4%) and of those, 166 (90.2%) met the inclusion criteria (142 from the Mayo Clinic and 24 from Vall d’Hebron) (Figure 1). The main demographics, interventions, and outcomes are summarized in Supplementary Tables S1 and S2, respectively.

#### Outcomes

Remission (BVAS/WG = 0) was achieved in 131 patients (78.9%) at 6 months and in a total of 136 patients (83.4%) during the follow-up. Relapses were documented in 41 patients (24.7%), and 31 of these (75.6%) were renal relapses. Dialysis was required in 106 patients (63.9%) throughout the entire follow-up. In 71 patients, dialysis was initiated within 4 weeks of presentation, and in 35 after 4 weeks; of the 71 patients, 27 were able to discontinue RRT. Of the 35 patients who started dialysis after week 4, most were women, MPO-ANCA positive, had lower SCr and higher eGFR at diagnosis (than patients who started dialysis within 4 weeks), received similar remission-induction treatment but were less frequently treated with PLEX or i.v. methylprednisolone, and only 5 patients were able to discontinue RRT. There were 60 patients who did not require dialysis, 46 were MPO-ANCA positive and 14 were PR3-positive, 56.7% of the patients had minimal or mild chronicity scores on kidney biopsies, and remission-induction treatment was similar with exception of PLEX (only 8 patients were treated with PLEX). A total of 16 patients died within 12 months. At the end of the follow-up (median of 4.0 years [IQR: 1.41–6.80]), a total of 59 patients (35.5%) had died, 33 patients (44.6%) were in dialysis. Death was attributed to active AAV in 16 patients. Patients included in the Vall d’Hebron cohort were more frequently MPO-ANCA positive and classified as MPA (Supplementary Table S3). Patients who started dialysis after 4 weeks were more frequently MPO-ANCA positive and had more features of chronicity with higher Mayo Clinic Chronicity score (Supplementary Table S4).

### Renal Function Recovery Within 12 Months (*n* = 145)

We analyzed renal recovery within 12 months in the 145 patients who were alive during the first year of follow-up and had eGFR data available at that time point (missing data in 5/150 patients). Eighty-one patients (55.9%) recovered renal function to an eGFR ≥15 ml/min per 1.73 m^2^ (Table 1). The patients who recovered kidney function within 12 months had higher eGFR (11.9 vs. 9.1 ml/min per 1.73 m^2^; *P* = 0.05) at diagnosis (Table 1). Most of these patients had kidney biopsies with minimal or mild chronicity changes (64.2% vs. 39.0%; *P* = 0.004), whereas patients who did not recover renal function had moderate or severe chronicity changes (60.9% vs. 35.8%; *P* = 0.004) (Table 1). These patients also showed lower chronicity score (4 vs. 6 points; *P* = 0.006). There were no differences in remission-induction treatment strategies, and a similar proportion of patients who recovered renal function had received PLEX compared to those who were not treated with PLEX (32.1% vs. 28.1%; *P* = 0.605) (Table 1; Supplementary Results and Supplementary Table S5).

**Table 1:** Clinical characteristics and outcomes of patients with antineutrophil cytoplasmic antibodies (ANCA)-associated vasculitis (AAV) with glomerulonephritis (AAV-GN) and eGFR <15 ml/min per 1.73 m^2^ at diagnosis according to renal recovery status to eGFR >15 within 12 months (*n* = 145)

Clinical characteristics and outcomes	No renal recovery *n* = 64 (44.1%)	Renal recovery *n* = 81 (55.9%)	*P-value*
Age at diagnosis, median (IQR), yr	65 (53.9–73.3)	68 (60.8–74.9)	0.138
Male, *n* (%)	32 (50.0)	41 (50.6)	0.941
AAV, *n* (%)			0.460
MPA	41 (64.1)	47 (58.0)	
GPA	41 (35.9)	34 (42.0)	
ANCA specificity (ELISA), *n* (%)			0.387
MPO	44 (68.8)	51 (63.0)	
PR3	20 (31.3)	30 (37.0)	
BVAS/WG at diagnosis, median (IQR)	7 (7–9)	7 (7–10)	0.475
Alveolar hemorrhage BVAS/WG at diagnosis, *n* (%)	15 (23.4)	15 (18.5)	0.468
Cardiovascular risk factors, *n* (%)			
Arterial hypertension	52 (81.3)	64 (79.0)	0.738
Diabetes mellitus	16 (25.0)	18 (22.2)	0.695
Dyslipidemia	27 (42.2)	39 (48.1)	0.474
BMI > 30 kg/m^2^	20 (31.6)	24 (29.6)	0.710
Laboratory findings			
Hemoglobin, mean (SD), g/dl	9.1 (1.95)	9.6 (1.40)	0.126
ESR, median (IQR), mm/1^st^h	60 (29–86)	86 (49–98)	0.015 a
SCr at diagnosis, median (IQR), mg/dl	5.1 (35–7.3)	4.4 (3.3–5.3)	0.020 a
eGFR at diagnosis of renal involvement, mean (SD), ml/min per 1.73 m^2^	9.1 (6.29–14.58)	11.9 (8.25–14.61)	0.050
MCCS, *n* (%)			
Minimal	10 (15.6)	16 (19.8)	0.520
Mild	15 (23.4)	36 (44.4)	0.009^a^
Moderate	24 (37.5)	18 (22.2)	0.044 a
Severe	15 (23.4)	11 (13.6)	0.124
MCCS, median (IQR), points	6 (3.0–7.0)	4 (2.0–6.0)	0.006 a
Intervention			
Remission-induction treatment, *n* (%)			0.683
Cyclophosphamide	45 (57.4)	39 (50.6)	
Rituximab	24 (39.3)	34 (44.2)	
Remission-induction adjuvant therapies, *n* (%)			
Plasma exchange therapy	18 (28.1)	26 (32.1)	0.605
i.v. methylprednisolone at induction remission	48 (75.0)	63 (77.8)	0.695
Outcomes, n (%)			
Relapse	9 (14.1%)	28 (34.6)	0.005 a
ESKD			
12 mo	50 (78.1)	0 (0.0)	-
Death	25 (39.1)	17 (21.0)	0.017 a
Infection	30 (46.9)	28 (34.6)	0.125
Time of FU after renal involvement, median (IQR), mo	51 (21.0–85.5)	56 (29.5–92.5)	0.300

AAV, ANCA-associated vasculitis; ANCA, antineutrophil cytoplasmic antibodies; BMI, body mass index; BVAS/WG, Birmingham Vasculitis Activity Score for Wegener's Granulomatosis; eGFR, estimated glomerular filtration rate; ELISA, enzyme-linked immunosorbent assay; ESKD, end-stage kidney disease; ESR, erythrocyte sedimentation rate; FU, follow-up; GPA, granulomatosis with polyangiitis; IQR, interquartile range; MCCS, Mayo Clinic Chronicity score; MPA, microscopic polyangiitis; MPO, myeloperoxidase; PLEX, plasma exchange; PR3, proteinase 3; SCr, serum creatinine.

a *P* ≤ 0.05, indicate statistical significance.

### ESKD Within 12 Months (*n* = 150)

During the first year of follow-up, 150 patients were alive and we could ascertain that they were not on dialysis. At 12 months, 50 patients (33.3%) were in ESKD (Table 2). At diagnosis, SCr was higher (5.8 vs. 4.1 mg/dl; *P* < 0.001) and eGFR was lower (7.9 vs. 12.1 ml/min per 1.73 m^2^; *P* < 0.0001) in patients who progressed to ESKD within 12 months (Table 2). These patients had a higher proportion of moderate or severe chronicity changes in kidney biopsies (64.0% vs. 39.0%; *P* = 0.010) and higher chronicity scores (6 vs. 4 points; *P* = 0.006) (Table 2). More patients who did not evolve to ESKD were treated with RTX (47.0% vs. 30.0%; *P* = 0.014). Relapses were more frequent in patients who did not evolve to ESKD within 12 months (31.0% vs. 12.0%; *P* = 0.011) (Table 2). At the end of the follow-up, 20 patients who were in ESKD at 12 months had died versus 23 patients without ESKD (40.0% vs. 23.0%; *P* = 0.030) (Table 2, Supplementary Results and Supplementary Table S6).

**Table 2:** Clinical characteristics and outcomes of patients with antineutrophil cytoplasmic antibodies (ANCA)-associated vasculitis (AAV) with glomerulonephritis (AAV-GN) and eGFR <15 ml/min per 1.73 m^2^ at diagnosis according to ESKD status within 12 months (*n* = 150)

Clinical characteristics and outcomes	No ESKD at 12 mo *n* = 100 (66.7%)	ESKD at 12 mo *n* = 50 (33.3%)	*P-value*
Age at diagnosis, median (IQR), yr	67 (59.5–74.6)	66 (54.2–74.7)	0.613
Male, *n* (%)	47 (47.0)	29 (58.0)	0.204
AAV, *n* (%)			0.407
MPA	59 (59.0)	33 (66.0)	
GPA	41 (41.0)	17 (34.0)	
ANCA specificity (ELISA), *n* (%)			0.715
MPO	65 (65.0)	34 (68.0)	
PR3	35 (35.0)	16 (32.0)	
BVAS/WG at diagnosis, median (IQR)	7 (7–9)	7 (7–9)	0.581
Alveolar hemorrhage BVAS/WG at diagnosis, *n* (%)	15 (15.0)	15 (30.0)	0.030^a^
Cardiovascular risk factors, *n* (%)			
Arterial hypertension	77 (77.0)	42 (84.0)	0.318
Diabetes mellitus	24 (24.0)	13 (26.0)	0.789
Dyslipidemia	48 (48.0)	20 (40.0)	0.354
BMI > 30 kg/m^2^	29 (29.0)	16 (32.0)	0.823
Laboratory findings			
Hemoglobin, mean (SD), g/dl	9.8 (1.57)	8.8 (1.67)	0.001^a^
ESR, median (IQR), mm/1^st^h	72 (39.8–97.0)	66 (44.5–93.0)	0.564
SCr at diagnosis, median (IQR), mg/dl	4.1 (3.2–5.1)	5.8 (4.1–8.7)	<0.0001^a^
eGFR at diagnosis, median (IQR), ml/min per 1.73 m^2^	12.1 (8.39–14.92)	7.9 (5.59–11.15)	<0.0001^a^
MCCS, *n* (%)			0.037^a^
Minimal	22 (22.0)	6 (12.0)	
Mild	39 (39.0)	12 (24.0)	
Moderate	23 (23.0)	20 (40.0)	
Severe	16 (16.0)	12 (24.0)	
MCCS, median (IQR), points	4.0 (2.0–6.0)	6.0 (3.0–7.3)	0.006^a^
Intervention			
Remission-induction treatment, *n* (%)			0.014^a^
Cyclophosphamide	48 (48.0)	32 (64.0)	
Rituximab	47 (47.0)	15 (30.0)	
Remission-induction adjuvant therapies, *n* (%)			
Plasma exchange therapy	26 (26.0)	18 (36.0)	0.205
i.v. methylprednisolone at induction remission	75 (75.0)	37 (74.0)	0.894
Outcomes, *n* (%)			
Relapse	31 (31.0)	6 (12.0)	0.011^a^
Dialysis			<0.0001^a^
Permanent	16 (16.0)	48 (96.0)	
Transient	31 (31.0)	0 (0.0)	
Death	23 (23.0)	20 (40.0)	0.030^a^
Infection	36 (36.0)	25 (50.0)	0.277
Time of FU after renal involvement, median (IQR), mo	54 (24.3–89.0)	51 (18.8–87.8)	0.599

AAV, ANCA-associated vasculitis; ANCA, antineutrophil cytoplasmic antibodies; BMI, body mass index; BVAS/WG, Birmingham Vasculitis Activity Score for Wegener's Granulomatosis; eGFR, estimated glomerular filtration rate; ELISA, enzyme-linked immunosorbent assay; ESKD, end-stage kidney disease; ESR, erythrocyte sedimentation rate; FU, follow-up; GPA, granulomatosis with polyangiitis; IQR, interquartile range; MCCS, Mayo Clinic Chronicity score; MPA, microscopic polyangiitis; MPO, myeloperoxidase; PLEX, plasma exchange; PR3, proteinase 3; SCr, serum creatinine.

a *P* ≤ 0.05, indicate statistical significance.

### Transient Versus Persistent Need for Dialysis (*n* = 71)

In 71 patients, dialysis was started within the first 4 weeks of the diagnosis. Dialysis was discontinued because of renal recovery in 27 (38.0%) whereas 44 patients (62.0%) remained dialysis-dependent (Table 3). Most of the patients who required transient dialysis had GPA, compared to MPA (63.0% vs. 38.6%; *P* = 0.046), had lower SCr (5.0 vs. 6.9 mg/dl; *P* = 0.011) and higher eGFR (10.3 vs. 7.6 ml/min per 1.73 m^2^; *P* = 0.009) at diagnosis. In addition, these patients had minimal or mild chronicity changes on kidney biopsies (74.0% vs. 40.9%; *P* = 0.007) and lower chronicity scores (3 vs. 5 points; *P* = 0.001) (Table 3). There were no differences in remission-induction treatment strategies between patients with GPA versus MPA, or in the proportion of patients who received PLEX (Table 3). In the group that remained dialysis-dependent, 23 patients died versus 5 patients in the group transiently dialyzed (52.3% vs. 18.5%; *P* = 0.005) (Table 3; Supplementary Results and Table 4). Of the 71 patients who started dialysis within the first 4 weeks, 11 patients died before 12 months (10 in dialysis; 1 had come off dialysis) and 4 patients received PLEX (all requiring permanent dialysis).

**Table 3:** Clinical characteristics and outcomes of patients with antineutrophil cytoplasmic antibodies (ANCA)-associated vasculitis (AAV) with glomerulonephritis (AAV-GN) and eGFR <15 ml/min per 1.73 m^2^ at diagnosis who started dialysis in the first 4 weeks of AAV-GN diagnosis according to dialysis status (*n* = 71)

Clinical characteristics and outcomes	Permanent *n* = 44 (62.0%)	Transient *n* = 27 (38.0%)	*P-value*
Age at diagnosis, median (IQR), yr	65 (55.3–73.2)	66 (60.0–72.0)	0.967
Male, *n* (%)	29 (65.9)	18 (66.7)	0.781
AAV, *n* (%)			0.046^a^
MPA	27 (61.4)	10 (37.0)	
GPA	17 (38.6)	17 (63.0)	
ANCA specificity (ELISA), *n* (%)			0.071
MPO	26 (59.1)	10 (37.0)	
PR3	18 (40.9)	17 (63.0)	
BVAS/WG at diagnosis, median (IQR)	8 (7–13)	8 (7–9)	0.201
Alveolar hemorrhage BVAS/WG at diagnosis, *n* (%)	13 (29.5)	8 (29.6)	0.994
Cardiovascular risk factors, *n* (%)			
Arterial hypertension	36 (81.8)	18 (66.7)	0.146
Diabetes mellitus	16 (36.4)	6 (22.2)	0.211
Dyslipidemia	19 (43.2)	15 (55.6)	0.311
BMI > 30 kg/m^2^	15 (34.1)	8 (29.6)	0.876
Laboratory findings			
Hemoglobin, mean (SD), g/dl	8.6 (1.64)	9.4 (1.58)	0.035^a^
ESR, median (IQR) mm/1^st^h	61 (41.5–93.0)	94 (50.8–98.8)	0.122
SCr at diagnosis, median (IQR), mg/dl	6.9 (4.8–9.3)	5.0 (2.7–7.0)	0.011^a^
eGFR at diagnosis, median (IQR), ml/min per 1.73 m^2^	7.6 (4.93–10.76)	10.3 (6.59–17.97)	0.033^a^
eGFR at 6 months, median (IQR), ml/min per 1.73 m^2^	12.1 (9.34–16.54)	32.8 (25.05–40.14)	<0.0001^a^
eGFR at 12 months, median (IQR), ml/min per 1.73 m^2^	9.1 (5.05–32.39)	30.3 (22.89–44.80)	0.021^a^
MCCS, *n* (%)			0.007^a^
Minimal	3 (6.8)	10 (37.0)	0.002^a^
Mild	15 (34.1)	10 (37.0)	0.805
Moderate	16 (36.4)	6 (22.2)	0.212
Severe	10 (22.7)	1 (3.7)	0.033^a^
MCCS, median (IQR), points	5 (3.0–7.0)	3 (1.0–5.0)	0.001^a^
Intervention			
Remission-induction treatment, *n* (%)			0.303
Cyclophosphamide	23 (52.3)	14 (51.9)	0.974
Rituximab	16 (36.4)	13 (48.1)	0.334
Remission-induction adjuvant therapies, *n* (%)			
Plasma exchange therapy	20 (45.5)	16 (59.3)	0.259
i.v. methylprednisolone at induction remission	39 (88.6)	25 (92.6)	0.587
Outcomes, *n* (%)			
Relapse	8 (18.2%)	11 (40.7)	0.037^a^
ESKD 12 mo	44 (100.0)	0 (0.0)	<0.0001^a^
Death	23 (52.3)	5 (18.5)	0.005^a^
Infection	21 (47.7)	14 (51.9)	0.567
Time of FU after renal involvement, median (IQR), mo	35 (4.0–85.5)	57 (24.0–82.0)	0.103

AAV, ANCA-associated vasculitis; ANCA, antineutrophil cytoplasmic antibodies; BMI, body mass index; BVAS/WG, Birmingham Vasculitis Activity Score for Wegener's Granulomatosis; eGFR, estimated glomerular filtration rate; ELISA, enzyme-linked immunosorbent assay; ESKD, end-stage kidney disease; ESR, erythrocyte sedimentation rate; FU, follow-up; GPA, granulomatosis with polyangiitis; IQR, interquartile range; MCCS, Mayo Clinic Chronicity score; MPA, microscopic polyangiitis; MPO, myeloperoxidase; PLEX, plasma exchange; PR3, proteinase 3; SCr, serum creatinine.

a *P* ≤ 0.05, indicate statistical significance.

**Table 4:** Univariable and multivariable logistic regression of the predictive factors for transient dialysis (*n* = 71)

Factors	*Univariable Analysis*			*Multivariable Analysis (adjusted to PLEX)*		
	OR	95% CI	*P-value*	OR	95% CI	*P-value*
Age ˂ 60 yr	0.933	0.336–2.591	0.895			
Male	1.034	0.375–2.853	0.948			
GPA	2.700	1.004–7.260	0.049^a^			
PR3	2.456	0.917–6.579	0.074			
Alveolar hemorrhage	1.004	0.351–2.868	0.994			
Hb at diagnosis ----	1.432	1.014–2.024	0.042^a^			
Erythrocyte sedimentation rate at diagnosis	1.013	0.995–1.031	0.171			
SCr at diagnosis	0.800	0.669–0.956	0.014^a^			
eGFR at diagnosis	1.069	1.001–1.141	0.046^a^			
MCCS						
Minimal	8.039	1.965–32.884	0.004^a^	6.138	1.389–27.118	0.017^a^
Mild	1.137	0.419–3.089	0.801			
Moderate	0.500	0.167–1.496	0.215			
Severe	0.131	0.016–1.087	0.060			
MCCS score	0.728	0.593–0.893	0.002^a^			
Berden						
Focal	6.875	1.906–24.798	0.003^a^	5.017	1.287–19.567	0.020^a^
Crescentic	1.329	0.494–3.573	0.573			
Mixed	0.276	0.081–0.938	0.039^a^			
Sclerotic	0.203	0.024–1.753	0.147			
Intervention						
Remission-induction treatment						
Rituximab (vs. CYC) ----	1.335	0.497–3.587	0.567			
Remission-induction adjuvant therapies						
Plasma exchange	1.745	0.661–4.606	0.261	1.650	0.548–4.965	0.373
i.v. methylprednisolone	1.603	0.288–8.905	0.590			

CI, confidence interval; CYC, Cyclophosphamide; eGFR, estimated glomerular filtration rate; Hb, hemoglobin; GPA, granulomatosis with polyangiitis; MCCS, Mayo Clinic Chronicity score; OR, odds ratio; PLEX, plasma exchange; PR3, proteinase 3; Scr, serum creatinine.

a *P* ≤ 0.05, indicate statistical significance.

### Predictors of Dialysis-Dependence at 12 Months in Patients who Started Dialysis in the First 4 Weeks (*n* = 71)

Patients who remained on dialysis at 12 months more often had higher SCr at diagnosis (IRR = 1.098; 95% CI: 1.026–1.175; *P* = 0.007), severe chronicity changes on kidney biopsies (IRR = 2.469; 95% CI: 1.205–5.061; *P* = 0.014), higher chronicity score (IRR = 1.178; 95% CI: 1.062–1.308; *P* = 0.002) and mixed glomerular involvement (IRR = 2.014; 95% CI: 1.093–3.710; *P* = 0.025). Minimal chronicity changes and focal glomerular involvement were protective for the achievement of this endpoint (IRR = 0.228; 95% CI: 0.070–0.741; *P* = 0.014 and IRR = 0.267; 95% CI: 0.095–0.749; *P* = 0.012, respectively).

Multivariable Cox regression showed that patients with higher SCr at diagnosis and with moderate or severe chronicity changes on biopsy were at a higher risk to evolve to ESKD within 12 months compared to patients with lower chronicity grades, irrespective of PLEX (Table 5).

**Table 5:** Univariable and multivariable Cox regression of the predictive factors for ESKD at 12 months in patients who started dialysis after 4 weeks of the diagnosis (*n* = 71)

Factors	*Univariable Analysis*			*Multivariable Analysis (adjusted to PLEX)*		
	IRR	95% CI	*P-value*	IRR	95% CI	*P-value*
Age < 60 yr	0.996	0.979–1.014	0.694			
Male	0.922	0.494–1.721	0.799			
GPA	0.565	0.308–1.038	0.066			
PR3	0.572	0.313–1.045	0.069			
Alveolar hemorrhage	0.945	0.494–1.807	0.864			
Hb at diagnosis ----	0.824	0.683–0.994	0.043^a^			
Erythrocyte sedimentation rate at diagnosis	0.991	0.981–1.001	0.094			
SCr at diagnosis	1.098	1.026–1.175	0.007^a^	1.086	1.005–1.173	0.037^a^
eGFR at diagnosis	0.951	0.904–1.001	0.054			
MCCS						
Minimal	0.228	0.070–0.741	0.014^a^	ref	ref	0.080
Mild	0.911	0.488–1.699	0.769	3.361	0.962–11.7373	0.057
Moderate	1.402	0.757–2.595	0.283	3.797	1.090–13.225	0.036^a^
Severe	2.469	1.205–5.061	0.014^a^	5.883	1.542–22.439	0.009^a^
MCCS score	1.178	1.062–1.308	0.002^a^			
Berden						
Focal	0.267	0.095–0.749	0.012^a^			
Crescentic	0.838	0.449–1.564	0.580			
Mixed	2.014	1.093–3.710	0.025^a^			
Sclerotic	1.952	0.862–4.421	0.109			
Intervention						
Remission-induction treatment						
Rituximab (vs. CYC) ----	0.980	0.517–1.857	0.950			
Remission-induction adjuvant therapies						
Plasma exchange	0.721	0.398–1.306	0.281	0.708	0.367–1.367	0.304
i.v. methylprednisolone	0.836	0.329–2.126	0.707			

CI, confidence interval; CYC, Cyclophosphamide; eGFR, estimated glomerular filtration rate; ESKD, end-stage kidney disease; Hb, hemoglobin; IRR, incidence rate ratio; GPA, granulomatosis with polyangiitis; MCCS, Mayo Clinic Chronicity score; PLEX, plasma exchange; PR3, proteinase 3; Scr, serum creatinine.

a *P* ≤ 0.05, indicate statistical significance.

### Plasma Exchange as Adjunct to Remission-Induction Therapy (*n* = 166)

**Figure 2:**
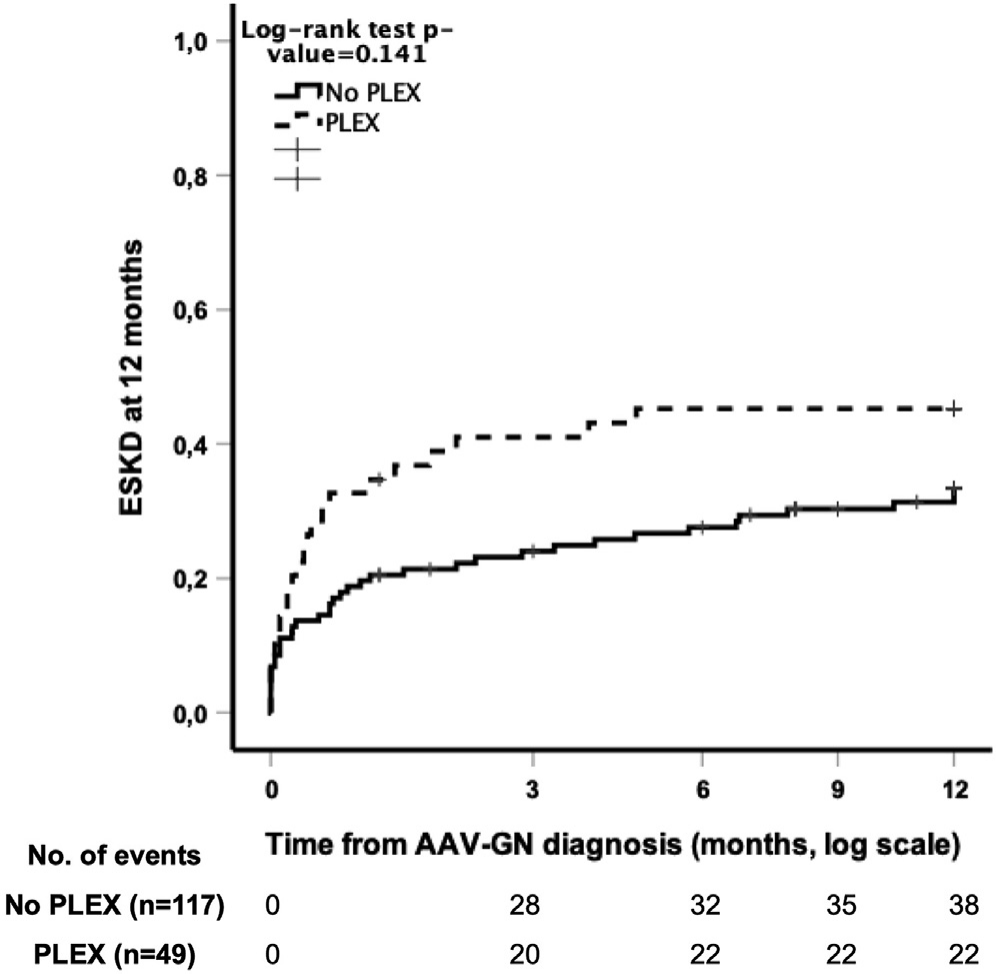
Kaplan-Meier plots of ESKD achieved within 12 months according to PLEX status in patients with eGFR <15 mL/min/1.73 m^2^ (*n* = 166). AAV, ANCA-associated vasculitis; ANCA, antineutrophil cytoplasmic antibodies, AAV-GN, AAV with glomerulonephritis, ESKD, end-stage kidney disease; PLEX, plasma exchange.

PLEX was added to standard immunosuppressive remission-induction therapy in 49 patients (29.5%). Patients who received PLEX also received concomitant i.v. methylprednisolone more frequently (93.9% vs. 70.1%; *P* = 0.001) (Supplementary Table S7). The median number of PLEX sessions was 7 (IQR: 5–9). Patients who received PLEX had lower median eGFR at diagnosis (8.0 vs. 11.1 ml/min per 1.73 m^2^; *P* = 0.035), required RRT more frequently (77.6% vs. 50.9%; *P* = 0.003) and had alveolar hemorrhage more frequently at diagnosis (36.7% vs. 12.3%; *P* < 0.001). The patients who received PLEX more frequently had minimal or mild chronicity scores than those who did not (66.3% vs. 44.5%; *P* = 0.006). Using Kaplan-Meier analysis, we detected no beneficial effect of adding PLEX in preventing ESKD at 12 months (*P* = 0.141), with the incidence of ESKD at 12 months being higher in patients who received PLEX (Figure 2) (Supplementary Table S7, Figure 1, and Supplementary Results).

### PLEX as Adjunct to Remission-Induction Therapy in Patients who Started Dialysis in the First 4 Weeks of Diagnosis (*n* = 71)

**Figure 3:**
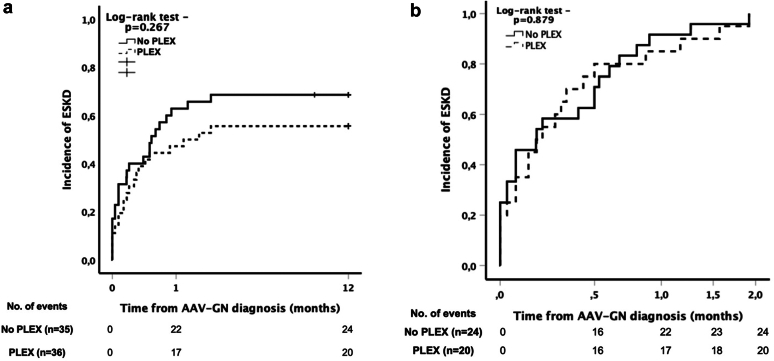
(a) Kaplan-Meier plots of dialysis initiation achieved within 12 months according to PLEX status in patients who started dialysis within 4 weeks of AAV-GN diagnosis (*n* = 71). (b) Kaplan-Meier plots of ESKD achieved at 2 months in patients under permanent dialysis according to PLEX status in patients who started dialysis within 4 weeks of AAV-GN diagnosis (*n* = 44). AAV, ANCA-associated vasculitis; ANCA, antineutrophil cytoplasmic antibodies, AAV-GN, AAV with glomerulonephritis, ESKD, end-stage kidney disease; PLEX, plasma exchange.

We analyzed the 71 patients who started dialysis in the first 4 weeks; 36 (50.7%) of these received PLEX, and 35 (49.3%) did not (Supplementary Table S8). The severity of kidney disease at diagnosis was similar between the groups (Supplementary Table S8). Patients who received PLEX more frequently presented with alveolar hemorrhage (41.7% vs. 17.1%; *P* = 0.024), and had more frequently been treated with CYC (72.2% vs. 31.4%; *P* = 0.004) and i.v. methylprednisolone (97.2% vs. 82.9%; *P* = 0.042) for remission-induction (Supplementary Table S8). There were no differences in demographic characteristics and outcomes (Supplementary Table S8). The Kaplan-Meier analysis showed that there were no differences in the time to ESKD when comparing patients who received PLEX to those who did not (Figure 3a); and that in patients who ultimately remained on dialysis permanently, the rate of progression to ESKD was similar when comparing patients who received PLEX with those who did not (*P* = 0.879) (Figure 3b).

### Remission-Induction Therapy in Patients who Started Dialysis in the First 4 Weeks of Diagnosis (*n* = 71)

In this subset of patients, 35 (53.0%) patients received CYC for remission-induction whereas 27 patients (40.9%) received RTX (Supplementary Results and Supplementary Tables S9– S11). There were no differences on the BVAS/WG scores, frequency of alveolar hemorrhage between groups, SCr and eGFR at diagnosis, and in the chronicity scores. PLEX was added to standard immunosuppressive remission-induction therapy more frequently in patients who received CYC (71.4% vs. 29.6%; *P* = 0.001); however, the use of i.v. methylprednisolone was similar between the groups (85.7% vs. 96.3%; *P* = 0.162). Patients who received CYC relapsed more (42.9% vs. 7.4%; *P* = 0.002). There were no differences between groups in the frequency of renal recovery, dialysis (permanent or transient), ESKD at 12 months or infections. Patients treated with CYC had worse survival, 18 (51.4%) died when compared with 7 (25.9%) in the RTX group (*P* = 0.042).

## Discussion

Our study showed that in patients with eGFR <15 ml/min per 1.73 m^2^, renal recovery to an eGFR >15 ml/min per 1.73 m^2^ at 12 months was dependent on lower SCr level, higher erythrocyte sedimentation rate, and minimal or mild chronicity changes on kidney biopsy at the time of AAV-GN diagnosis. Furthermore, in patients who started dialysis within 4 weeks of diagnosis, the presence of minimal chronicity changes and focal involvement were the main determinants of dialysis discontinuation, regardless of the use of PLEX. Conversely, ESKD at 12 months was predicted by lower levels of hemoglobin, higher SCr, and presence of moderate or severe chronicity changes on kidney biopsy at diagnosis. In the patients who started dialysis early in the course of the disease, there was an increased risk of remaining dialysis-dependent for those with moderate or severe chronicity changes on biopsy when compared to lower degrees of chronicity changes, even when adjusted for PLEX, with no benefit of PLEX in terms of renal function recovery or prevention of progression to ESKD at 12 months, in our cohort. In contrast, patients who started dialysis after 4 weeks most likely had a smoldering disease because they were more frequently MPO-ANCA, had lower SCr level at diagnosis but higher chronicity (Supplementary Table S4). In decade analyses, renal recovery and survival improved in patients with severe AAV (Supplementary Table S10).

Recently, a systematic review of all 9 randomized controlled trials published by July 2020 evaluated the effects of PLEX on AAV outcomes at 12 months, using data derived from studies conducted over a 40-year period.^22^ The authors’ concluded that the addition of PLEX to standard remission-induction in AAV probably reduced the risk of ESKD at 12 months (relative risk [RR]: 0.62; 95% CI: 0.39–0.98).^22^ In the subgroup analyses of patients with SCr ≤5.7 mg/dl versus SCr >5.7 mg/dl, there were no differences in the RR for ESKD at 12 months (RR: 0.70; 95% CI: 0.43–1.12 vs. RR: 0.83; 95% CI: 0.56–1.01; *P* = 0.55).^22^ The meta-analysis showed that PLEX was associated with an estimated absolute RR for ESKD at 12 months of 16.0% (4.2% to 23.6%) for those at high risk or SCr>5.7 mg/dl (high certainty of important effects). These findings were interpreted as supportive of PLEX in patients with AAV and high risk of progression to ESKD.^33^ These results differ from the PEXIVAS trial that found no benefit of adding PLEX to standard remission-induction therapy in patients with eGFR <50 ml/min per 1.73 m^2^ or with SCr >5.6 mg/dl.^24^ Of note, the proportion of patients who received PLEX and required hemodialysis in the PEXIVAS study was similar when compared to the MEPEX trial, suggesting a role for improvement in global kidney disease standard-of-care in modulating the benefit of adding PLEX to remission-induction treatment.^24^ Our results, obtained in a cohort that most closely reflets current clinical practice, mirror those of the PEXIVAS trial in that the addition of PLEX did not contribute to renal recovery or to avoid progression to ESKD. Indeed, the prognosis of patients included in this cohort with severe AAV-GN was dependent on the level of SCr at presentation and on the kidney biopsies findings, suggesting that at similar levels of eGFR, patients with higher SCr may have more severe kidney disease. Furthermore, the impact of novel treatments (i.e., C5a inhibitors) in avoiding progression to ESKD was not taken into consideration in the meta-analysis.^24^ Cortazar *et al.*^34^ recently showed that among patients with baseline eGFR ≤20 ml/min per 1.73 m^2^ in the ADVOCATE (Avacopan for the Treatment of ANCA-Associated Vasculitis) trial, eGFR improved more in the avacopan group when compared to the prednisone group (mean change of eGFR 16.1 vs. 7.7 ml/min per 1.73 m^2^; *P* = 0.003; increase in eGFR in ≥2-fold in 40.7% vs. 13.0%; *P* = 0.030). The integration of these data on the treatment of patients with eGFR <15 ml/min per 1.73 m^2^ is likely to change the standard-of-care of patients with severe kidney disease.^35^ Importantly, there are no data from randomized controlled trials in this population and our results were retrieved from the largest cohort of patients with AAV and severe kidney disease with eGFR <15 ml/min per 1.73 m^2^ representing the better evidence regarding their management.

Models of response to PLEX based on kidney histopathology have been proposed.^23^ Nezam *et al.*^23^ developed a prediction model to estimate in which patients the use of PLEX could lower the probability of death or RRT by 12 months using data from 248 patients in which PLEX was not recommended and compared with 177 patients in which PLEX was recommended. Patients with MPA, MPO-ANCA, higher SCr, crescentic and sclerotic classes, and higher Brix score, were more frequent in the PLEX recommended group. In this study, the absolute risk difference for death or RRT at 12 months was -4.8% (95% CI, -14.9% to 5.3%) in the PLEX not recommended group vs. -15.9% (95% CI, -29.4% to -2.5%) in the PLEX recommended group.^23^ However, it was pointed out that this corresponds to a very similar degree of RR decrease in both groups (4.8%/13.9% = 34.5% vs. 15.9%/51.6% = 30.8%),^36^ concluding that the effect of PLEX was similar in both groups. Indeed, in our cohort (*n* = 166), patients treated with PLEX had more frequently mild chronicity score on kidney biopsies suggesting that even in the presence of favorable factors (i.e., less severe disease on kidney biopsies) treatment with PLEX did not translate to better outcomes at 12 months, with SCr and moderate and severe chronicity changes as the main predictors of ESKD at 12 months regardless of treatment with PLEX. Moreover, when we analyzed the patients who were started on dialysis at the time of AAV-GN diagnosis (*n* = 71), we found that patients who had GPA, were PR3-ANCA positive, or had a SCr <5.7 mg/dl at the time of diagnosis, with focal involvement or minimal or mild chronicity changes were more likely to discontinue dialysis, regardless of the treatment with PLEX, further questioning the utility of PLEX in patients with eGFR <15 ml/min per 1.73 m^2^.

This study has limitations inherent to its retrospective design. First, our cohort consists mainly of a Midwestern US White population with predominantly Scandinavian and Northern European and Spaniard ethnic backgrounds. Therefore, the results may not be generalizable. Second, treatment assignments were not protocol-driven. Third, the dosing and tapering of glucocorticoids was not protocolized. Fourth, the nature of the patients observed, and pace of real clinic practice might differ when compared to clinical trials. Fifth, there were inherent differences in clinical practice patterns between USA and Spain that we were not able to account for in our analysis, such as the time to receive remission-induction treatment, combined immunosuppression for remission-induction and PLEX methodologies. We also acknowledge that in our study, SCr at inception ranged between 3.4 and 6.3 mg/dl (IQR) and more than 50% of the patients had a Scr of <4.7 mg/dl, which is considerably lower than the levels of 5.7 or 5.6 mg/dl used for analysis in MEPEX and in a subanalysis of PEXIVAS. However, and in contrast to MEPEX, we did not focus only on patients who were randomized to receive PLEX or not, but rather on kidney disease severity based on eGFR <15 ml/min per 1.73 m^2^ or need for dialysis.

Despite these limitations, this is the largest observational cohort of patients with AAV-GN and eGFR <15 ml/min per 1.73 m^2^ providing a detailed analysis of clinical characteristics and outcomes in response to different treatments in real clinical practice. Even though the treatment of these patients did not follow a strictly standardized protocol, there is homogeneity in the care of these patients by a group of experts in AAV, which followed consistent patterns and decisions. In addition, all the patients underwent kidney biopsy, making this the largest biopsy-proven cohort of patients of AAV-GN and eGFR <15 ml/min per 1.73 m^2^. Another advantage is that the patients compared were all naturally matched by disease severity (due to the inclusion criteria eGFR <15 ml/min per 1.73 m^2^) in a cohort where the proportion of ESKD at 12 months was constant along the decades. This allows the inference that the success of interventions along the decades were similar, precluding the need to use complicated methodologies of analysis that would be limited by the sample size validating the robustness of the analysis performed. Furthermore, overall improvement on the rates of ESKD over the past decades have been mainly attributed to early diagnosis rather than to therapeutic interventions,^4^^,^^5^^,^^10^ meaning that the differences in the standard-of-care between decades do not account for substantial heterogeneity. Finally, treatment with PLEX was used in patients with the same severity of kidney disease regardless of the group comparison performed. The fact that the patients differ between groups is not related to the severity of the kidney disease but to a random allocation to a treatment strategy according to other factors related with disease presentation.

In conclusion, our study suggests that response to treatment in severe AAV-GN depends on SCr and histologic findings on kidney biopsies at the time of diagnosis, regardless of PLEX. Patients with minimal or mild chronicity changes will have higher probability of renal recovery within 12 months, may be able to discontinue dialysis, whereas patients with higher SCr and moderate or severe chronicity changes at diagnosis will be more likely to remain or progress to dialysis within 12 months. Large multicenter randomized controlled trials in this patient population are needed to further explore our results.

## Disclosure

MCM is partially supported through a Vasculitis Clinical Research Consortium-Vasculitis Foundation Fellowship. MJS reports honorarium for conferences, consulting fees, and advisory boards from AstraZeneca, NovoNordisk, Esteve, Vifor, Bayer, Mundipharma, Ingelheim Lilly, Jansen, ICU Medical, Travere Therapeutics, GE Healthcare, and Boehringer. KJW has received clinical trial support from Eli Lilly, GSK and Kiniksa; he received consulting fees and honoraria from Chemocentryx. US reports advisory board fees from ChemoCentryx, AstraZeneca, and Boehringer Ingelheim; and research grant support from Bristol Myer Squibb, Glaxo Smith Kline, AstraZeneca, and Genentech. FCF has received unrestricted research grants from Roche/Genentech. All the other authors declared no competing interests.

## Supplementary Material

**Supplementary Methods.**

**Supplementary Results.**

**Table S1.** Demographic and clinical characteristics of patients with antineutrophil cytoplasmic antibodies (ANCA)-associated vasculitis (AAV) with glomerulonephritis (AAV-GN) and estimated glomerular filtration rate <15 ml/min per 1.73 m^2^ at diagnosis (*n* = 166).

**Table S2.** Outcomes of patients with antineutrophil cytoplasmic antibodies (ANCA)-associated vasculitis (AAV) with glomerulonephritis (AAV-GN) and estimated glomerular filtration rate <15 ml/min per 1.73 m^2^ at diagnosis (*n* = 166).

**Table S3.** Clinical characteristics and outcomes of patients with antineutrophil cytoplasmic antibodies (ANCA)-associated vasculitis (AAV) with glomerulonephritis (AAV-GN) and estimated glomerular filtration rate <15 ml/min per 1.73 m^2^ according to the center (*n* = 166).

**Table S4.** Clinical characteristics and outcomes of patients with antineutrophil cytoplasmic antibodies (ANCA)-associated vasculitis (AAV) with glomerulonephritis (AAV-GN) and estimated glomerular filtration rate <15 ml/min per 1.73 m^2^ stratified by patients who started dialysis after 4 weeks versus within 4 weeks.

**Table S5.** Univariable and multivariable logistic regression of the predictive factors for renal recovery at 12 months.

**Table S6.** Univariable and multivariable logistic regression of the predictive factors for end-stage kidney disease at 12 months.

**Table S7.** Univariable and multivariable logistic regression of the predictive factors for transient dialysis.

**Table S8.** Univariable and multivariable Cox regression of the predictive factors for end-stage kidney disease at 12 months in patients who started dialysis after 4 weeks of the diagnosis.

**Table S9.** Clinical characteristics and outcomes of patients with antineutrophil cytoplasmic antibodies (ANCA)-associated vasculitis (AAV) with glomerulonephritis (AAV-GN) and estimated glomerular filtration rate <15 ml/min per 1.73 m^2^ at diagnosis according to remission-induction treatment.

**Table S10.** Clinical characteristics and outcomes of patients with antineutrophil cytoplasmic antibodies (ANCA)-associated vasculitis (AAV) with glomerulonephritis (AAV-GN) and estimated glomerular filtration rate <15 ml/min per 1.73 m^2^ according to the decade.

**Table S11.** Clinical characteristics and outcomes of patients with antineutrophil cytoplasmic antibodies (ANCA)-associated vasculitis (AAV) with glomerulonephritis (AAV-GN) and estimated glomerular filtration rate <15 ml/min per 1.73 m^2^ and/or at dialysis within the first 4 weeks at diagnosis according to remission-induction treatment.
