# Supplementary material for: Predictive Factors of Renal Recovery and Progression to End-Stage Kidney Disease in Patients With Antineutrophil Cytoplasmic Autoantibody-Associated Vasculitis With Severe Kidney Disease
Source: Kidney Int Rep. 2024 Mar 6;9(5):1284–97. doi: 10.1016/j.ekir.2024.02.1431 (PMC11068975; doi:10.1016/j.ekir.2024.02.1431)
Supplement: Supplementary File (PDF) [file mmc1.pdf]

Supplementary Material

Supplementary Methods

Statistical analysis. (i) Logistic regression. Variables were considered for the multivariable logistic regression models if they occurred before the development of the outcome of interest, had <10% of missing values, had p values less than 0.05 in the univariable analysis and were clinically plausible. The final model was determined using both clinical and statistical criteria, taking into consideration collinearity, interaction and the number of patients who experienced the outcome of interest. Some of the continuous variables were categorized with cut-offs determined according to pre-established guidelines or clinical practice. The odds ratio (OR) with 95% confidence intervals (95%CI) were reported when appropriate. The results derived from these analyses are described on the *Supplementary Material*.

(ii) The Kaplan Meier method and Cox proportional hazards regression models. Patients who did not experience a relevant outcome were censored in survival analyses but excluded in reporting of cumulative incidences of outcomes at specific times if they were not observed for the entire duration. We excluded an observation from the analysis if the underlying outcome could not be ascertained. A multivariable Cox proportional hazards regression model was used to assess the predictive factors of renal recovery and ESKD at 12 months. We adjusted all our multivariate models for the use of PLEX regardless of the significance obtained in the univariate model.

Supplementary Results

Renal function recovery at 12 months (n= 145). Multivariable logistic regression analysis showed that SCr at diagnosis, erythrocyte sedimentation rate and minimal/mild

chronicity changes on biopsies were the most important predictive factors for renal recovery, even when adjusted for treatment with PLEX (*Supplementary Table S5*).

ESKD within 12 months (n=150) Multivariable logistic regression analysis showed that higher SCr, lower hemoglobin at diagnosis and moderate to severe chronicity grading on kidney biopsy were the most important predictive factors for progression to ESKD at 12 months, even when adjusted for the treatment with PLEX (*Supplementary Table S6*).

Transient versus persistent need for dialysis (n=71). Multivariable logistic regression analysis showed that minimal chronicity changes or focal involvement were the most important predictors of transient dialysis, even when adjusted for the treatment with PLEX (*Supplementary Table S7*).

PLEX as adjunct to remission-induction therapy in patients that started dialysis in the first 4 weeks of diagnosis (n=71). We then further analyzed the 36 patients treated with PLEX among the 71 patients who underwent dialysis within 4 weeks. Of these 36 patients, 16 patients were dialysis-independent at 12 months and 20 remained dialysis-dependent at 12 months. Patients who became dialysis-independent following PLEX more frequently had diagnosis of GPA (62.5% vs. 35.0%, p=0.101), PR3-ANCA (65.0% vs. 40.0%,p=0.180), a SCr <5.7 mg/dL (68.8% vs. 25.0%,p=0.009), and had minimal/mild chronicity changes on kidney biopsies (81.3% vs. 55.0%,p=0.097). Conversely, of the 35 patients that were not treated with PLEX, 11 were dialysis-independent at 12 months, whereas 24 remained dialysis-dependent. Patients who became dialysis-independent without PLEX more often had a diagnosis of GPA diagnosis (63.6% vs. 36.4%,p=0.227), were PR3-ANCA positive (63.6% vs. 36.4%,p=0.227), and had minimal/mild chronicity changes on the kidney biopsy (63.6% vs. 29.2%,p=0.053). The

proportion of patients with SCr <5.7 mg/dL was not different between patients who became dialysis-independent vs. who remained on dialysis (63.5% vs. 58.3%,p=0.766) in this subset.

Remission-induction therapy in patients with eGFR <15 mL/min/1.73 m^2 and/or dialysis dependent (n=166). In the 166 patients, 81 (51.9%) patients received cyclophosphamide (CYC) for remission-induction whereas 69 (44.2%) patients received rituximab (RTX) (*Supplementary Table S9).* In the remaining 10 patients, 3 (1.8%) were treated with MMF and in 7 (4.2%) patients only prednisone was used for remission-induction treatment mainly due to contraindication to CYC (*Supplementary Table S1*). When comparing CYC vs. RTX, we excluded from the analysis the 6 patients from the Vall d'Hebron cohort that have received both CYC and RTX. There were no differences on the BVAS/WG scores, frequency of alveolar hemorrhage between groups, and SCr and eGFR at diagnosis. A higher proportion of patients scored as moderate in the chronicity score in the RTX group (55.1% vs. 39.5%,p=0.024). PLEX was added to standard immunosuppressive remission-induction therapy more frequently in patients who received CYC (42.0% vs. 15.9%,p=0.001) whereas IV methylprednisolone was more commonly added in patients treated with RTX (91.3% vs. 67.9%,p<0.001). Patients who received CYC relapsed more (33.3% vs. 15.9%,p=0.015). There were no differences between groups in the frequency of renal recovery, dialysis (permanent or transient), ESKD at 12 months or infections. Patients treated with CYC had worse survival, 37 (45.7%) died when compared with 17 (24.6%) in the RTX group (p=0.007). In the decade analysis, treatment choices reflected the standard of care with the incremental use of RTX and discontinuation of addition of PLEX in the more recent years (*Supplementary Table S10).* Importantly, renal recovery and survival improved along the years whereas the ESKD frequency remained constant between decades, highlighting this unmet need.

Supplementary Tables

Supplementary Table S1 - Demographic and clinical characteristics of patients Anti-Neutrophil Cytoplasmic Antibodies (ANCA) associated vasculitis (AAV) patients with glomerulonephritis (AAV-GN) and eGFR < 15 mL/min/1.73 m^2 at diagnosis (n=166).

Supplementary Table S2 - Outcomes of patients with Anti-Neutrophil Cytoplasmic Antibodies (ANCA) associated vasculitis (AAV) patients with glomerulonephritis (AAV-GN) and eGFR < 15 mL/min/1.73 m^2 at diagnosis (n=166).

Supplementary Table S3 - Clinical characteristics and Outcomes with Anti-Neutrophil Cytoplasmic Antibodies (ANCA) associated vasculitis (AAV) patients with glomerulonephritis (AAV-GN) and eGFR < 15 mL/min/1.73 m2 according with the center (n=166).

Supplementary Table S4 - Clinical characteristics and Outcomes with Anti-Neutrophil Cytoplasmic Antibodies (ANCA) associated vasculitis (AAV) patients with glomerulonephritis (AAV-GN) and eGFR < 15 mL/min/1.73 m2 stratified by patients who started dialysis after 4 weeks vs. within 4 weeks.

Supplementary Table S5 – Univariable and Multivariable logistic regression of the predictive factors for renal recovery at 12 months.

Supplementary Table S6 – Univariable and Multivariable logistic regression of the predictive factors for ESKD at 12 months.

Supplementary Table S7 – Clinical characteristics and Outcomes with Anti-Neutrophil Cytoplasmic Antibodies (ANCA) associated vasculitis (AAV) patients with glomerulonephritis (AAV-GN) and eGFR < 15 mL/min/1.73 m^2 at diagnosis according with the status of treatment with plasma exchange (PLEX) (n=166).

Supplementary Table S8 – Clinical characteristics and Outcomes with Anti-Neutrophil Cytoplasmic Antibodies (ANCA) associated vasculitis (AAV) patients with glomerulonephritis (AAV-GN) and eGFR < 15 mL/min/1.73 m^2 at diagnosis who started dialysis in the first 4 weeks of AAV-GN diagnosis according with PLEX status (n=71).

Supplementary Table S9 - Clinical characteristics and Outcomes with Anti-Neutrophil Cytoplasmic Antibodies (ANCA) associated vasculitis (AAV) patients with glomerulonephritis (AAV-GN) and eGFR < 15 mL/min/1.73 m^2 at diagnosis according with remission-induction treatment.

Supplementary Table S10 - Clinical characteristics and Outcomes with Anti-Neutrophil Cytoplasmic Antibodies (ANCA) associated vasculitis (AAV) patients with glomerulonephritis (AAV-GN) and eGFR < 15 mL/min/1.73 m2 according with the decade.

Supplementary Table S11 - Clinical characteristics and Outcomes with Anti-Neutrophil Cytoplasmic Antibodies (ANCA) associated vasculitis (AAV) patients with glomerulonephritis (AAV-GN) and eGFR < 15 mL/min/1.73 m^2 and/or at dialysis within the first 4 weeks at diagnosis according with remission-induction treatment.

Supplementary Table S1 - Demographic and clinical characteristics of patients Anti-Neutrophil Cytoplasmic Antibodies (ANCA) associated vasculitis (AAV) patients with glomerulonephritis (AAV-GN) and eGFR < 15 mL/min/1.73 m^2 at diagnosis (n=166).

	AAV-GN with eGFR < 15 mL/min/1.73 m^2 at diagnosis n = 166
Age at renal involvement diagnosis, median (IQR) years	67 (58-75)
Male, n (%)	88 (53.0)
Disease presentation, n (%)	
AAV new diagnosis	147 (88.6)
AAV relapse	19 (11.4)
AAV, n (%)	
MPA	102 (61.4)
GPA	64 (38.6)
ANCA specificity (ELISA), n (%)	
MPO	109 (65.7)
PR3	57 (34.3)
BVAS/WG at renal involvement diagnosis, median (IQR)	7 (7-9)
Alveolar hemorrhage, n (%)	32 (19.3)
Cardiovascular risk factors, n (%)	
Arterial hypertension	130 (78.3)
Diabetes mellitus	43 (25.9)
Dyslipidemia	76 (45.8)
BMI > 30 Kg/m^2	51 (30.3)
Laboratory findings	
Hemoglobin, mean (sd) g/dL	9.3 (1.67)
ESR, median (IQR) mm/1sth	70 (44.2-95.0)
SCr at diagnosis, median (IQR) mg/dL	4.7 (3.4-6.3)
eGFR at diagnosis, median (IQR), mL/min/1.73m^2	10.5 (7.3-14.1)
eGFR at 6 months, median (IQR), mL/min/1.73m^2	25.4 (16.6-37.6)
eGFR at 12 months, median (IQR), mL/min/1.73m^2	25.1 (17.0-41.2)
Biopsy proven, n (%)	166 (100.0)
MCCS, n (%)	
Minimal	28 (16.9)
Mild	57 (34.3)
Moderate	50 (30.1)
Severe	31 (18.7)
MCCS, median (IQR), points	4 (2.8 – 7.0)
Intervention	
Remission-induction treatment, n (%)	

Cyclophosphamide	84 (50.6)
Rituximab	72 (43.4)
PDN only	7 (4.2)
Mycophenolate mofetil	3 (1.8)
Glucocorticoids, n (%)	
IV methylprednisolone	128 (77.1)
Oral prednisone	38 (22.9)
Plasma exchange therapy, n (%)	49 (29.5)
Maintenance treatment, n (%)	
Rituximab	39 (23.5)
Azathioprine	35 (21.1)
Mycophenolate mofetil	34 (20.5)
Prednisone	16 (9.6)
Cyclophosphamide	7 (4.2)
Methotrexate	1 (0.6)
No remission-maintenance or impossible to determine	21 (12.6)

Abbreviations: AAV - antineutrophil cytoplasmic antibody associated vasculitis; ANCA - anti-neutrophil cytoplasmic antibody; BVAS/WG - Birmingham vasculitis activity score for Wegener granulomatosis; eGFR – estimated glomerular filtration rate; ESR - erythocyte sedimentation rate; GPA - ganulomatosis with polyangiitis; IQR - interquartile range; IV – intravenous; MCCS – Mayo Clinic Chronicity Score; MPA - microscopic polyangiitis; MPO - myeloperoxidase; n- number; PLEX – Plasma exchange; PR3 - proteinase 3; SCr - serum creatinine.

Supplementary Table S2 - Outcomes of patients with Anti-Neutrophil Cytoplasmic Antibodies (ANCA) associated vasculitis (AAV) patients with glomerulonephritis (AAV-GN) and eGFR < 15 mL/min/1.73 m^2 at diagnosis (n=166).

	AAV-GN with eGFR < 15 mL/min/1.73 m^2 at diagnosis<br>n = 166
**Outcomes**	
Remission	136 (83.4)
Relapse	41 (24.7)
Death	59 (35.5)
Renal, n (%)	
ESKD	
12 months	50 (33.3)
Dialysis	106 (63.9)
Dialysis within 4 weeks of AAV-GN diagnosis	71 (42.8)
Transient Dialysis	32 (19.3)
Permanent Dialysis	74 (44.6)
Renal function recovery to an eGFR > 15 mL/min/1.73m^2	
12 months	70 (42.2)
Infection within 12 months, n (%)	66 (39.8)
Time of FU after renal involvement, median (IQR) years	4.0 (1.41 – 6.8)

# - "Total" corresponds to the number of the event documented in all time of follow-up.

Abbreviations: AAV – anti-neutrophil cytoplasmic antibody associated vasculitis; eGFR – estimated glomerular filtration rate; FU – follow-up; IQR – interquartile range; KF – kidney failure; n - number.

Supplementary Table S3 - Clinical characteristics and Outcomes with Anti-Neutrophil Cytoplasmic Antibodies (ANCA) associated vasculitis (AAV) patients with glomerulonephritis (AAV-GN) and eGFR < 15 mL/min/1.73 m2 according with the center (n=166).

	Mayo Clinic n = 142 (85.5%)	Vall d'Hebron n = 24 (14.5%)	p-value
Age at diagnosis, median (IQR) years	67 (59.8 – 75.9)	69 (57.3 – 76.0)	0.845
Male, n (%)	73 (51.4)	15 (62.5)	0.314
AAV, n (%)			**0.017**
MPA	82 (57.7)	20 (83.3)	
GPA	60 (42.3)	4 (16.7)	
ANCA specificity (ELISA), n (%)			**0.049**
MPO	89 (62.7)	20 (83.3)	
PR3	53 (37.3)	4 (16.7)	
BVAS/WG at diagnosis, median (IQR)	7 (7-9)	7 (7-10)	0.429
Alveolar hemorrhage BVAS/WG at diagnosis, n (%)	26 (18.3)	6 (25.0)	0.442
Cardiovascular risk factors, n (%)			
Arterial hypertension	113 (79.6)	17 (70.8)	0.336
Diabetes mellitus	39 (27.5)	4 (16.4)	0.264
Dyslipidemia	68 (47.9)	8 (33.3)	0.186
BMI > 30 kg/m^2	48 (33.8)	3 (12.5)	0.110
Laboratory findings			
Hemoglobin, mean (sd) g/dL	9.3 (1.65)	9.2 (1.80)	0.935
ESR, median (IQR) mm/1^{st}h	67 (43.0 – 93.0)	96 (44.0 – 120.0)	0.069
SCr at diagnosis, median (IQR) mg/dL	4.6 (3.3 – 5.8)	5.0 (3.6 – 8.1)	0.157
eGFR at diagnosis of renal involvement, mean (sd), mL/min/1.73m^2	10.6 (7.43 - 13.89)	9.4 (5.96 – 14.88)	0.538
MCCS, n (%)			0.129
Minimal	26 (18.3)	2 (8.3)	0.227
Mild	51 (35.9)	6 (25.0)	0.285
Moderate	38 (26.8)	12 (50.0)	**0.022**
Severe	27 (19.0)	4 (16.7)	0.785
MCCS, median (IQR), points	4 (2.0 - 7.0)	6 (3.0 - 7.0)	0.100
Remission-induction treatment, n (%)			0.170
Cyclophosphamide	74 (56.1)	7 (38.9)	
Rituximab	58 (43.9)	11 (61.1)	
Remission-induction adjuvant therapies, n (%)			
Plasma exchange therapy	38 (26.8)	11 (45.8)	0.058
IV methylprednisolone at induction remission	106 (74.6)	22 (91.7)	0.066
Outcomes, n (%)			
Relapse	33 (23.2)	8 (33.3)	0.289
Renal recovery	69 (51.9)	12 (54.5)	0.817
Dialysis			0.649
Permanent	63 (44.4)	11 (45.8)	
Transient	26 (18.3)	6 (25.0)	
ESKD			
12 months	50 (35.2)	10 (41.7)	0.543
Death	51 (35.9)	8 (33.3)	0.807
Infection	28 (19.7)	6 (25.0)	0.311
Time of FU after renal involvement, median (IQR) months	43 (16.0 – 82.5)	56 (17.5 – 79.3)	0.963

Supplementary Table S4 - Clinical characteristics and Outcomes with Anti-Neutrophil Cytoplasmic Antibodies (ANCA) associated vasculitis (AAV) patients with glomerulonephritis (AAV-GN) and eGFR < 15 mL/min/1.73 m^2 stratified by patients who started dialysis after 4 weeks vs. within 4 weeks.

	Dialysis after 4 weeks n = 35 (33.0%)	Dialysis within 4 weeks n = 71 (67.0%)	p-value
Age at diagnosis, median (IQR) years	65 (50.0 – 73.3)	66 (58.0 – 73.0)	0.362
Male, n (%)	11 (31.4)	47 (66.2)	**0.001**
AAV, n (%)			0.295
MPA	22 (62.9)	37 (52.1)	
GPA	13 (37.1)	34 (47.9)	
ANCA specificity (ELISA), n (%)			**0.009**
MPO	27 (77.1)	36 (50.7)	
PR3	8 (22.9)	35 (49.3)	
BVAS/WG at diagnosis, median (IQR)	7 (7-9)	9 (7-10)	0.094
Alveolar hemorrhage BVAS/WG at diagnosis, n (%)	6 (17.1)	21 (29.6)	0.167
Cardiovascular risk factors, n (%)			
Arterial hypertension	29 (82.9)	54 (76.1)	0.424
Diabetes mellitus	8 (22.9)	22 (31.0)	0.382
Dyslipidemia	17 (48.6)	34 (47.9)	0.947
BMI > 30 kg/m^2	15 (42.9)	23 (32.4)	0.552
Laboratory findings			
Hemoglobin, mean (sd) g/dL	10.0 (1.93)	8.8 (1.66)	0.218
ESR, median (IQR) mm/1sth	67 (31.5 – 90.3)	70 (45.0 – 97.0)	0.065
SCr at diagnosis, median (IQR) mg/dL	3.4 (2.3 – 4.6)	5.7 (4.2 – 8.6)	0.165
eGFR at diagnosis of renal involvement, mean (sd), mL/min/1.73m^2	10.6 (7.43 - 13.89)	9.4 (5.96 – 14.88)	0.182
MCCS, n (%)			
Minimal	6 (17.1)	13 (18.3)	0.883
Mild	7 (20.0)	25 (35.2)	0.109
Moderate	12 (34.3)	22 (31.0)	0.732
Severe	10 (28.6)	11 (15.5)	0.112
MCCS, median (IQR), points	6 (3.0 - 9.0)	4 (3.0 - 7.0)	**0.001**
Remission-induction treatment, n (%)			0.782
Cyclophosphamide	18 (52.9)	35 (53.0)	
Rituximab	15 (44.1)	27 (40.9)	
Remission-induction adjuvant therapies, n (%)			
Plasma exchange therapy	5 (14.3)	36 (50.7)	**<0.0001**
IV methylprednisolone at induction remission	21 (60.0)	64 (90.1)	**<0.0001**
Outcomes, n (%)			
Relapse	9 (25.7)	19 (26.8)	0.909
Renal recovery	9 (25.7)	25 (36.2)	0.280
Dialysis			**0.012**
Permanent	30 (85.7)	44 (62.0)	
Transient	5 (14.3)	27 (38.0)	
ESKD			
12 months	14 (40.0)	44 (52.0)	**0.033**
Death	11 (31.4)	28 (39.4)	0.421
Infection	4 (11.4)	18 (25.4)	0.311
Time of FU after renal involvement, median (IQR) months	60 (32.0 – 92.0)	41 (12.0 – 82.0)	0.529

Supplementary Table S5 – Univariable and Multivariable logistic regression of the predictive factors for renal recovery at 12 months (n=145).

	Univariable Analysis			*Multivariable Analysis (adjusted to PLEX)*		
	OR	95% CI	*p-value*	OR	95% CI	*p-value*
Age < 60 years	1.719	0.853-3.467	0.130			
Male	1.025	0.532-1.974	0.941			
GPA	1.684	0.894-3.173	0.107			
PR3	1.294	0.646-2.593	0.467			
Alveolar hemorrhage	0.742	0.332-1.661	0.469			
Hb at diagnosis ----	1.172	0.945-1.454	0.148			
Erythrocyte sedimentation rate at diagnosis	1.014	1.002-1.025	**0.021**	1.015	1.002-1.028	**0.028**
SCr at diagnosis	0.829	0.729-0.942	**0.004**	0.793	0.654-0.962	**0.018**
eGFR at diagnosis	0.995	0.973-1.018	0.682			
MCCS						
Minimal	1.329	0.558-3.168	0.521	4.225	1.726-10.340	**0.002**
Mild	2.613	1.265-5.399	**0.009**	4.225	1.726-10.340	**0.002**
Moderate	0.476	0.230-0.987	**0.046**	ref.	-	-
Severe	0.513	0.217-1.212	0.128	ref.	-	-
MCCS score	0.848	0.752-0.956	**0.007**			
Intervention						
Remission-induction treatment						
Rituximab (vs. CYC) ----	1.271	0.635-2.544	0.498			
Remission-induction adjuvant therapies						
Plasma exchange	1.208	0.590-2.475	0.605	1.805	0.596-5.467	0.296
IV methylprednisolone	1.167	0.540-2.522	0.695			

Supplementary Table S6 – Univariable and Multivariable logistic regression of the predictive factors for ESKD at 12 months (n=166).

	Univariable Analysis			*Multivariable Analysis (adjusted to PLEX)*		
	OR	95% CI	*p-value*	OR	95% CI	*p-value*
Age < 60 years	0.892	0.449-1.772	0.892			
Male	1.558	0.820-2.959	0.176			
GPA	1.268	0.657-2.445	0.479			
PR3	0.933	0.478-1.820	0.715			
Alveolar hemorrhage	2.429	1.073-5.496	**0.033**			
Hb at diagnosis ----	0.694	0.553-0.872	**0.002**	0.733	0.566-0.947	**0.018**
Erythrocyte sedimentation rate at diagnosis	0.996	0.985-1.007	0.457			
SCr at diagnosis	1.399	1.201-1.628	**<0.0001**	1.365	1.143-1.631	**0.001**
eGFR at diagnosis	0.928	0.876-0.984	**0.012**			
MCCS						
Minimal	ref.	-	-	ref.	-	-
Mild	1.584	0.537-4.526	0.415	ref.	-	-
Moderate	3.123	1.073-4.644	**0.035**	2.952	1.307-6.66	**0.002**
Severe	3.020	0.715-3.84	0.059	4.400	1.716-11.282	**0.002**
MCCS score	1.159	1.035-1.330	0.011			
Intervention						
Remission-induction treatment						
Rituximab (vs. CYC) ----	0.618	0.315-1.214	0.163			
Remission-induction adjuvant therapies						
Plasma exchange	1.694	0.856-3.354	0.130	0.920	0.356-2.376	0.864
IV methylprednisolone	1.116	0.522-2.387	0.778			

Supplementary Table S7 – Clinical characteristics and Outcomes with Anti-Neutrophil Cytoplasmic Antibodies (ANCA) associated vasculitis (AAV) patients with glomerulonephritis (AAV-GN) and eGFR < 15 mL/min/1.73 m^2 at diagnosis according with the status of treatment with plasma exchange (PLEX) (n=166).

	No PLEX n = 117 (70.5%)	PLEX n = 49 (29.5%)	*p-value*
Age at diagnosis, median (IQR) years	68 (57-77)	65 (59-72)	0.158
Male, n (%)	57 (48.7)	31 (63.3)	0.087
AAV, n (%)			0.277
MPA	75 (64.1)	27 (55.1)	
GPA	42 (35.9)	22 (44.9)	
ANCA specificity (ELISA), n (%)			0.064
MPO	82 (70.1)	27 (55.1)	
PR3	35 (29.9)	22 (44.9)	
BVAS/WG at diagnosis, median (IQR)	7 (7-9)	8 (7-10)	**0.013**
Alveolar hemorrhage BVAS/WG at diagnosis, n (%)	14 (12.0)	18 (36.7)	**<0.0001**
Cardiovascular risk factors, n (%)			
Arterial hypertension	95 (81.2)	35 (71.4)	0.164
Diabetes mellitus	32 (27.4)	11 (22.4)	0.511
Dyslipidemia	54 (46.2)	22 (44.9)	0.882
BMI > 30 kg/m^2	37 (31.6)	14 (28.6)	0.895
Laboratory findings			
Hemoglobin, mean (sd) g/dL	9.5 (1.66)	8.8 (1.60)	**0.019**
ESR, median (IQR) mm/1sth	65 (40.8-91.5)	93 (45.1-112.5)	**0.048**
SCr at diagnosis, median (IQR) mg/dL	4.1 (3.30-5.20)	5.8 (4.40-8.65)	**<0.0001**
eGFR at diagnosis, median (IQR), mL/min/1.73m^2	11.1 (7.90 – 14.71)	8.0 (5.75-12.37)	**0.003**
MCCS, n (%)			**0.015**
Minimal	20 (17.1)	8 (16.3)	0.900
Mild	32 (27.4)	25 (51.0)	**0.004**
Moderate	38 (32.5)	12 (24.5)	0.307
Severe	27 (23.1)	4 (8.2)	**0.025**
MCCS, median (IQR), points	5 (3-7)	3 (2-6)	
Remission-induction treatment, n (%)			**0.009**
Cyclophosphamide	49 (41.9)	35 (71.4)	**<0.0001**
Rituximab	59 (50.4)	13 (26.5)	**0.005**
Remission-induction adjuvant therapies, n (%)			
IV methylprednisolone at induction remission	82 (70.1)	46 (93.9)	**0.001**
Maintenance treatment, n (%)			0.732
Azathioprine	22 (18.8)	13 (26.5)	
Mycophenolate mofetil	21 (17.9)	13 (26.5)	
Rituximab	29 (24.8)	9 (18.4)	
Prednisone	12 (10.3)	4 (8.2)	
Outcomes, n (%)			
Relapse	23 (19.7)	18 (36.7)	**0.020**
ESKD			
12 months	38 (32.5)	22 (44.9)	0.129
Dialysis			**<0.0001**
Transient	15 (12.8)	17 (34.7)	**0.001**
Permanent	50 (42.7)	24 (49.0)	0.458
Renal recovery			
12 months	45 (48.4)	26 (56.5)	0.367
Death	40 (34.2)	19 (38.8)	0.573
Infection	44 (37.6)	22 (44.9)	0.778
Time of FU after renal involvement, median (IQR) months	35 (13.0-71.0)	60 (36.5 – 90.0)	**0.027**

Supplementary Table S8 – Clinical characteristics and Outcomes with Anti-Neutrophil Cytoplasmic Antibodies (ANCA) associated vasculitis (AAV) patients with glomerulonephritis (AAV-GN) and eGFR < 15 mL/min/1.73 m^2 at diagnosis who started dialysis in the first 4 weeks of AAV-GN diagnosis according with PLEX status (n=71).

	No PLEX<br>n = 35 (48.3%)	PLEX<br>n = 36 (50.7%)	*p-value*
Age at diagnosis, median (IQR) years	66 (56.0-73.2)	64 (58.7-72.9)	0.593
Male, n (%)	22 (62.9)	25 (69.4)	0.557
AAV, n (%)			0.909
MPA	18 (51.4)	19 (52.8)	
GPA	17 (48.6)	19 (52.8)	
ANCA specificity (ELISA), n (%)			0.904
MPO	18 (51.4)	18 (50.0)	
PR3	17 (48.6)	18 (50.0)	
BVAS/WG at diagnosis, median (IQR)	8 (7-9)	9 (7-12)	0.312
Alveolar hemorrhage BVAS/WG at diagnosis, n (%)	6 (17.1)	15 (41.7)	**0.024**
Cardiovascular risk factors, n (%)			
Arterial hypertension	27 (77.1)	27 (75.0)	0.832
Diabetes mellitus	12 (34.3)	10 (27.8)	0.553
Dyslipidemia	17 (48.6)	17 (47.2)	0.909
BMI > 30 kg/m^2	14 (40.0)	9 (25.0)	0.346
Laboratory findings			
Hemoglobin, mean (sd) g/dL	9.0 (1.61)	8.8 (1.72)	0.740
ESR, median (IQR) mm/1sth	66 (45.5-93.0)	93 (45.0-112.0)	0.298
SCr at diagnosis, median (IQR) mg/dL	5.0 (3.7-7.4)	5.9 (4.9-9.2)	0.169
eGFR at diagnosis, median (IQR), mL/min/1.73m^2	8.9 (6.26-14.65)	7.8 (5.19-11.56)	0.260
MCCS, n (%)			0.098
Minimal	6 (17.1)	7 (19.4)	0.803
Mild	8 (22.9)	17 (47.2)	**0.033**
Moderate	13 (37.1)	9 (25.0)	0.274
Severe	8 (22.9)	3 (8.3)	0.092
MCCS, median (IQR), points	5 (3.0-8.0)	4 (3.0-6.0)	0.098
Intervention			
Remission-induction treatment, n (%)			
Cyclophosphamide	11 (31.4)	26 (72.2)	**0.004**
Rituximab	20 (57.1)	9 (25.0)	0.116
Remission-induction adjuvant therapies, n (%)			
IV methylprednisolone at induction remission	29 (82.9)	35 (97.2)	**0.042**
Outcomes, n (%)			
Relapse	6 (17.1)	13 (36.1)	0.071
ESKD<br>12 months	24 (68.6)	20 (55.6)	0.259
Dialysis			0.259
Transient	11 (31.4)	16 (44.4)	
Permanent	24 (68.6)	20 (55.6)	
Death	14 (40.0)	14 (38.9)	0.924
Infection	19 (54.3)	16 (44.4)	0.833
Time of FU after renal involvement, median (IQR) years	24 (8.0-61.0)	61 (32.0-92.3)	**0.019**

Supplementary Table S9 - Clinical characteristics and Outcomes with Anti-Neutrophil Cytoplasmic Antibodies (ANCA) associated vasculitis (AAV) patients with glomerulonephritis (AAV-GN) and eGFR < 15 mL/min/1.73 m^2 and/or at dialysis at diagnosis according with remission-induction treatment (n=166).

	Cyclophosphamide<br>n = 81 (51.9%)	Rituximab<br>n = 69 (44.2%)	*p-value*
Age at diagnosis, median (IQR) years	67 (54.0 – 75.7)	67 (60.0 – 77.7)	0.321
Male, n (%)	43 (53.1)	37 (53.6)	0.607
AAV, n (%)			0.124
MPA	44 (54.3)	46 (66.7)	

GPA	37 (45.7)	46 (45.1)	
ANCA specificity (ELISA), n (%)			0.415
MPO	50 (61.7)	47 (68.1)	
PR3	31 (38.3)	22 (31.9)	
BVAS/WG at diagnosis, median (IQR)	7 (7-9)	7 (7-8)	0.214
Alveolar hemorrhage BVAS/WG at diagnosis, n (%)	15 (18.5)	14 (20.3)	0.784
Cardiovascular risk factors, n (%)			
Arterial hypertension	60 (74.1)	55 (79.7)	0.416
Diabetes mellitus	14 (17.3)	23 (33.3)	**0.023**
Dyslipidemia	33 (40.7)	36 (52.2)	0.161
BMI > 30 kg/m^2	22 (27.2)	23 (33.3)	0.279
Laboratory findings			
Hemoglobin, mean (sd) g/dL	9.2 (1.54)	9.3 (1.85)	0.935
ESR, median (IQR) mm/1sth	78 (50.8 – 95.8)	51 (29.5 – 93.0)	0.051
SCr at diagnosis, median (IQR) mg/dL	4.9 (3.4 – 6.1)	4.5 (3.4 - 6.3)	0.642
eGFR at diagnosis of renal involvement, mean (sd), mL/min/1.73m^2	9.7 (7.2 - 13.69)	11.3 (7.24 – 14.60)	0.518
MCCS, n (%)			
Minimal	16 (19.8)	11 (15.9)	0.542
Mild	30 (40.7)	20 (29.0)	0.133
Moderate	19 (23.5)	28 (40.6)	**0.024**
Severe	13 (16.0)	10 (14.5)	0.792
MCCS, median (IQR), points	4 (2.0-7.0)	5 (3.0-7.0)	0.281
Remission-induction adjuvant therapies, n (%)			
Plasma exchange therapy	34 (42.0)	11 (15.9)	**0.001**
IV methylprednisolone at induction remission	55 (67.9)	63 (91.3)	**<0.001**
Outcomes, n (%)			
Relapse	27 (33.3%)	11 (15.9)	**0.015**
Renal recovery	36 (44.4)	25 (36.2)	0.745
Dialysis			0.736
Permanent	38 (46.9)	28 (40.6)	
Transient	15 (18.5)	14 (20.3)	
ESKD			
12 months	32 (39.5)	21 (30.4)	0.247
Death	37 (45.7)	17 (24.6)	**0.007**
Infection	42 (43.8)	24 (34.3)	0.064
Time of FU after renal involvement, median (IQR) months	61 (30.0 – 125.5)	29 (12.0 – 52.0)	**<0.0001**

Supplementary Table S10 - Clinical characteristics and Outcomes with Anti-Neutrophil Cytoplasmic Antibodies (ANCA) associated vasculitis (AAV) patients with glomerulonephritis (AAV-GN) and eGFR < 15 mL/min/1.73 m2 according with the decade.

	1996-2005<br>n = 38 (22.9%)	2006-2015<br>n=61 (36.7%)	2016-2021<br>n = 67 (40.4%)	p-value
Age at diagnosis, median (IQR) years	66 (48.6 – 78.9)	69 (61.0 – 75.3)	67 (60.0 – 73.0)	0.812
Male, n (%)	18 (47.4)	34 (55.7)	36 (53.7)	0.711
AAV, n (%)				0.117
MPA	19 (50.0)	43 (70.5)	40 (59.7)	
GPA	19 (50.0)	28 (29.5)	27 (40.3)	
ANCA specificity (ELISA), n (%)				0.400
MPO	24 (63.2)	44 (72.1)	42 (61.2)	
PR3	14 (36.8)	17 (27.9)	26 (38.8)	
BVAS/WG at diagnosis, median (IQR)	7 (7-9)	7 (7-10)	7 (7-10)	0.809

Alveolar hemorrhage BVAS/WG at diagnosis, n (%)	3 (17.9)	11 (18.0)	18 (26.9)	0.058
Cardiovascular risk factors, n (%)				
Arterial hypertension	38 (78.9)	45 (73.5)	55 (82.1)	0.519
Diabetes mellitus	7 (18.4)	12 (19.7)	24 (35.8)	0.056
Dyslipidemia	12 (31.6)	29 (47.5)	35 (52.2)	0.117
BMI > 30 kg/m^2	9 (23.7)	18 (29.5)	24 (35.8)	0.234
Laboratory findings				
Hemoglobin, mean (sd) g/dL	9.2 (1.50)	9.6 (1.51)	9.1 (1.90)	0.198
ESR, median (IQR) mm/1sth	72 (47.0 – 93.0)	70 (39.0 - 102.0)	63 (44.0 – 120.0)	0.983
SCr at diagnosis, median (IQR) mg/dL	4.5 (3.4 – 5.3)	4.3 (3.3 – 6.1)	4.7 (3.4 – 7.0)	0.600
eGFR at diagnosis of renal involvement, mean (sd), mL/min/1.73m^2	10.5 (7.91 - 14.01)	9.7 (7.01 – 14.08)	10.7 (7.00 – 14.54)	0.852
MCCS, n (%)				0.129
Minimal	7 (18.4)	10 (16.4)	11 (16.4)	0.958
Mild	12 (31.6)	24 (39.3)	21 (31.3)	0.585
Moderate	11 (28.9)	15 (24.6)	24 (35.8)	0.378
Severe	8 (21.2)	12 (19.7)	11 (16.4)	0.816
MCCS, median (IQR), points	4 (2.0 - 7.0)	4 (3.0-7.0)	5 (2.0 - 7.0)	0.939
Remission-induction treatment, n (%)				**<0.0001**
Cyclophosphamide	35 (97.2)	39 (69.6)	7 (10.9)	
Rituximab	1 (2.8)	15 (26.8)	53 (82.8)	
Remission-induction adjuvant therapies, n (%)				
Plasma exchange therapy	5 (13.2)	28 (45.9)	16 (23.9)	**0.001**
IV methylprednisolone at induction remission	20 (52.6)	48 (78.7)	60 (89.6)	**<0.0001**
Outcomes, n (%)				
Relapse	14 (36.8)	14 (23.0)	13 (19.4)	0.127
Renal recovery	8 (44.4)	28 (66.7)	24 (80.0)	**0.041**
Dialysis				0.378
Permanent	18 (47.4)	29 (47-5)	27 (40.3)	
Transient	5 (13.2)	9 (14.8)	18 (26.9)	
ESKD				
12 months	11 (28.9)	17 (27.9)	16 (23.9)	0.767
Death	23 (60.5)	23 (37.7)	13 (19.4)	**<0.0001**
Infection	5 (13.2)	11 (18.0)	18 (26.9)	0.311
Time of FU after renal involvement, median (IQR) months	65 (16.0 – 151.5)	61 (35.0 – 93.5)	25 (12.0 – 54.0)	**<0.0001**

Supplementary Table S11 - Clinical characteristics and Outcomes with Anti-Neutrophil Cytoplasmic Antibodies (ANCA) associated vasculitis (AAV) patients with glomerulonephritis (AAV-GN) and eGFR < 15 mL/min/1.73 m^2 and/or at dialysis within the first 4 weeks at diagnosis according with remission-induction treatment (n=71).

	Cyclophosphamide n = 35 (53.0%)	Rituximab n = 27 (40.9%)	*p-value*
Age at diagnosis, median (IQR) years	66 (47.0 – 74.9)	65 (60.0 – 72.0)	0.787
Male, n (%)	26 (74.3)	17 (63.0)	0.338
AAV, n (%)			0.798
MPA	18 (51.4)	13 (48.1)	
GPA	17 (48.6)	14 (51.9)	
ANCA specificity (ELISA), n (%)			0.442
MPO	19 (54.3)	12 (44.4)	
PR3	16 (45.7)	15 (55.6)	
BVAS/WG at diagnosis, median (IQR)	7 (7-9)	7 (7-10)	0.429
Alveolar hemorrhage BVAS/WG at diagnosis, n (%)	10 (28.6)	9 (33.3)	0.687
Cardiovascular risk factors, n (%)			
Arterial hypertension	25 (71.4)	21 (77.8)	0.571
Diabetes mellitus	7 (20.0)	12 (44.4)	**0.038**
Dyslipidemia	15 (42.9)	16 (59.3)	0.200
BMI > 30 kg/m^2	7 (20.0)	13 (48.1)	0.051
Laboratory findings			
Hemoglobin, mean (sd) g/dL	8.9 (1.63)	8.7 (1.87)	0.467
ESR, median (IQR) mm/1sth	91 (49.5 – 100.5)	60 (33.5 – 93.0)	0.096
SCr at diagnosis, median (IQR) mg/dL	5.7 (4.2 - 8.2)	5.2 (4.1 – 8.7)	0.904
eGFR at diagnosis of renal involvement, mean (sd), mL/min/1.73m^2	8.5 (5.92 - 13.59)	8.0 (5.63 – 12.42)	0.893

MCCS, n (%)			
Minimal	7 (20.0)	5 (18.5)	0.884
Mild	15 (42.9)	8 (29.6)	0.285
Moderate	9 (25.7)	11 (40.7)	0.209
Severe	4 (11.4)	3 (11.1)	0.969
MCCS, median (IQR), points	4 (3.0 - 7.0)	5 (2.0 - 7.0)	0.775
Remission-induction adjuvant therapies, n (%)			
Plasma exchange therapy	25 (71.4)	8 (29.6)	**0.001**
IV methylprednisolone at induction remission	30 (85.7)	26 (96.3)	0.162
Remission-maintenance treatment, n (%)			
Azathioprine	9 (25.7)	0 (0.0)	-
Mycophenolate mofetil	12 (34.3)	1 (3.7)	0.004
No maintenance	5 (14.3)	11 (40.7)	0.020
Rituximab	4 (11.4)	11 (40.7)	0.008
Oral prednisone only	2 (5.7)	4 (14.8)	0.233
Outcomes, n (%)			
Relapse	15 (42.9)	2 (7.4)	**0.002**
Renal recovery	13 (37.1)	9 (33.3)	0.756
Dialysis			0.736
Permanent	21 (60.0)	16 (59.3)	
Transient	14 (40.0)	11 (40.7)	
ESKD			
12 months	21 (60.0)	16 (59.3)	0.953
Death	18 (51.4)	7 (25.9)	**0.042**
Infection	17 (48.6)	13 (48.1)	0.531
Time of FU after renal involvement, median (IQR) months	68 (37.0 – 127.0)	20 (8.0 – 43.0)	0.775
